# cryoTIGER: deep-learning based tilt interpolation generator for enhanced reconstruction in cryo electron tomography

**DOI:** 10.1038/s42003-025-08961-5

**Published:** 2025-10-09

**Authors:** Tomáš Majtner, Jan Philipp Kreysing, Maarten W. Tuijtel, Sergio Cruz-León, Jiasui Liu, Gerhard Hummer, Martin Beck, Beata Turoňová

**Affiliations:** 1 Department of Molecular Sociology, Max Planck Institute of Biophysics, Frankfurt am Main, Germany; 2 IMPRS on Cellular Biophysics, Frankfurt am Main, Germany; 3 Department of Theoretical Biophysics, Max Planck Institute of Biophysics, Frankfurt am Main, Germany; 4 Institute of Biophysics, Goethe University Frankfurt, Frankfurt am Main, Germany; 5 Institute of Biochemistry, Goethe University Frankfurt, Frankfurt am Main, Germany

**Keywords:** Cryoelectron tomography, Image processing

## Abstract

Cryo-electron tomography enables the visualization of macromolecular complexes within native cellular environments but is limited by incomplete angular sampling and the maximal electron dose that biological specimens can be exposed to. Here, we developed cryoTIGER (Tilt Interpolation Generator for Enhanced Reconstruction), a computational workflow leveraging deep learning-based frame interpolation to generate intermediate tilt images. By interpolating between tilt series projections, cryoTIGER improves angular sampling, leading to enhanced 3D reconstructions, more refined particle localization, and improved segmentation of cellular structures. We evaluated our interpolation workflow on diverse datasets and compared its performance against non-interpolated data. Our results demonstrate that deep learning-based interpolation improves image quality and structural recovery. The presented cryoTIGER framework offers a computational alternative to denser sampling during tilt series acquisition, paving the way for enhanced cryo-ET workflows and advancing structural biology research.

## Abstract (web summary)

Deep learning-based interpolation using cryoTIGER enhances angular sampling in cryoET, improving 3D reconstructions, particle localization, and structural recovery without increasing electron dose.

## Introduction

Cryo-electron tomography (cryo-ET) has revolutionized our ability to visualize macromolecular complexes within their native cellular environments. At the heart of cryo-ET is the tilt series acquisition process, where a biological specimen is imaged at incremental tilt angles to generate a series of two-dimensional (2D) projections. These projections are then computationally reconstructed into a three-dimensional (3D) volume called a tomogram, providing insights into the structural organization of cellular components^1^.

The acquisition setup requires optimizing the interplay of multiple parameters at once to obtain tilt series with desired qualities. The most crucial parameters are the total electron dose imposed on the sample, the tilt range, and the tilt increment. Since biological samples are highly sensitive to radiation damage, excessive electron dose will degrade the sample, compromising the integrity of the structural information^2,3^. The tilt range determines the effective thickness of the sample during tilting and, more importantly, the extent of the missing wedge (i.e., the angular space with missing signal)^4–6^.

Finally, the tilt increment, the angular step between successive projections, directly influences the completeness of angular sampling and thus the completeness of the 3D reconstruction. The relationship between the angular sampling and the resolution beyond which the signal content becomes incomplete is described by the Crowther criterion^7^. Smaller increments provide more complete angular sampling, thereby enhancing the contrast and visibility of smaller features^4,8,9^. To maintain a reasonable tilt range, one has to either increase the total electron dose or decrease the dose per tilt, both of which complicate the subsequent processing by lowering the signal-to-noise ratio (SNR) for each image^10^. Conversely, larger increments allow for a higher electron dose per tilt but lead to poorer angular sampling and stronger artifacts in the tomograms^5,11^.

In standard practice, tilt series are typically acquired at increments of two or three degrees with a tilt range ±60 degrees^12^. This setup has proven itself well-suited for obtaining high-resolution structures using the subtomogram averaging (STA) workflow in which multiple instances of the same complex are localized within tomograms and then aligned and averaged together^10^. The aligning and averaging of randomly oriented particles effectively extends the angular sampling and thus reduces the missing wedge in the obtained structure.

A crucial step of STA is reliable particle localization, which remains challenging, especially for smaller complexes. The most common localization methods are template matching^13–15^, deep-learning (DL) based approaches^16–19^, and surface-based localization that is used for pleomorphic assemblies^20–23^. While the negative impact of the missing wedge on the depiction of features that are elongated perpendicularly to the beam direction has been well described^24,25^, the extent to which the incomplete angular sampling between the tilts negatively influences those methods remains understudied. Consequently, most of the research is focused on filling the missing wedge^26–28^ while the incomplete angular sampling between tilts has not been systematically explored.

When looking at angular sampling from the perspective of computer vision, one wants to synthesize intermediate images between a pair of input tilts with a certain motion. In general, it is possible to address this with traditional methods based on linear or tricubic interpolation^29^ or more advanced DL-based image interpolation techniques^30^. The latter leverage the power of convolutional neural networks (CNNs)^31^, recurrent neural networks (RNNs)^32^, and generative adversarial networks (GANs)^33^ to learn representations of image content and spatial relationships. DL models are trained on large datasets, allowing them to capture a wide range of textures and motions displayed in the field of view. To the best of our knowledge, none of these interpolation methods have been applied so far to generate additional images within cryo-ET tilt series.

Here we present cryoTIGER: Tilt Interpolation Generator for Enhanced Reconstruction for cryo-ET, which computationally reduces the angular spacing by interpolating between the neighboring images within the tilt series. We adopted a DL-based frame interpolation algorithm called FILM^34^ for the cryo-ET workflow and trained models on multiple datasets, providing sufficient diversity in acquisition parameters and cellular content. We evaluated different models and compared them to linear interpolation as well as to non-interpolated data. The results showed that in comparison to non-interpolated data, DL-based interpolation yielded better outcomes in most use cases. Our study underlines the importance of more complete angular sampling between the tilts and provides a computational solution that reduces the need to physically acquire datasets with exceedingly dense angular sampling.

## Results

### Adaptation to the cryo-ET workflow

To interpolate artificially generated tilt images in between experimentally acquired tilts, we choose frame interpolation for large motion (FILM)^34^. The FILM network is a UNet-style architecture with 5 encoder-decoder levels and skip connections, using strided convolutions for downsampling and bilinear upsampling in the decoder. The network contains ~24.5 million trainable parameters. The training requires ~1 GB of GPU memory for a batch size of 4. Memory usage scales linearly with input resolution and batch size. For more technical details, we refer the reader to the original work.

**Fig. 1:**
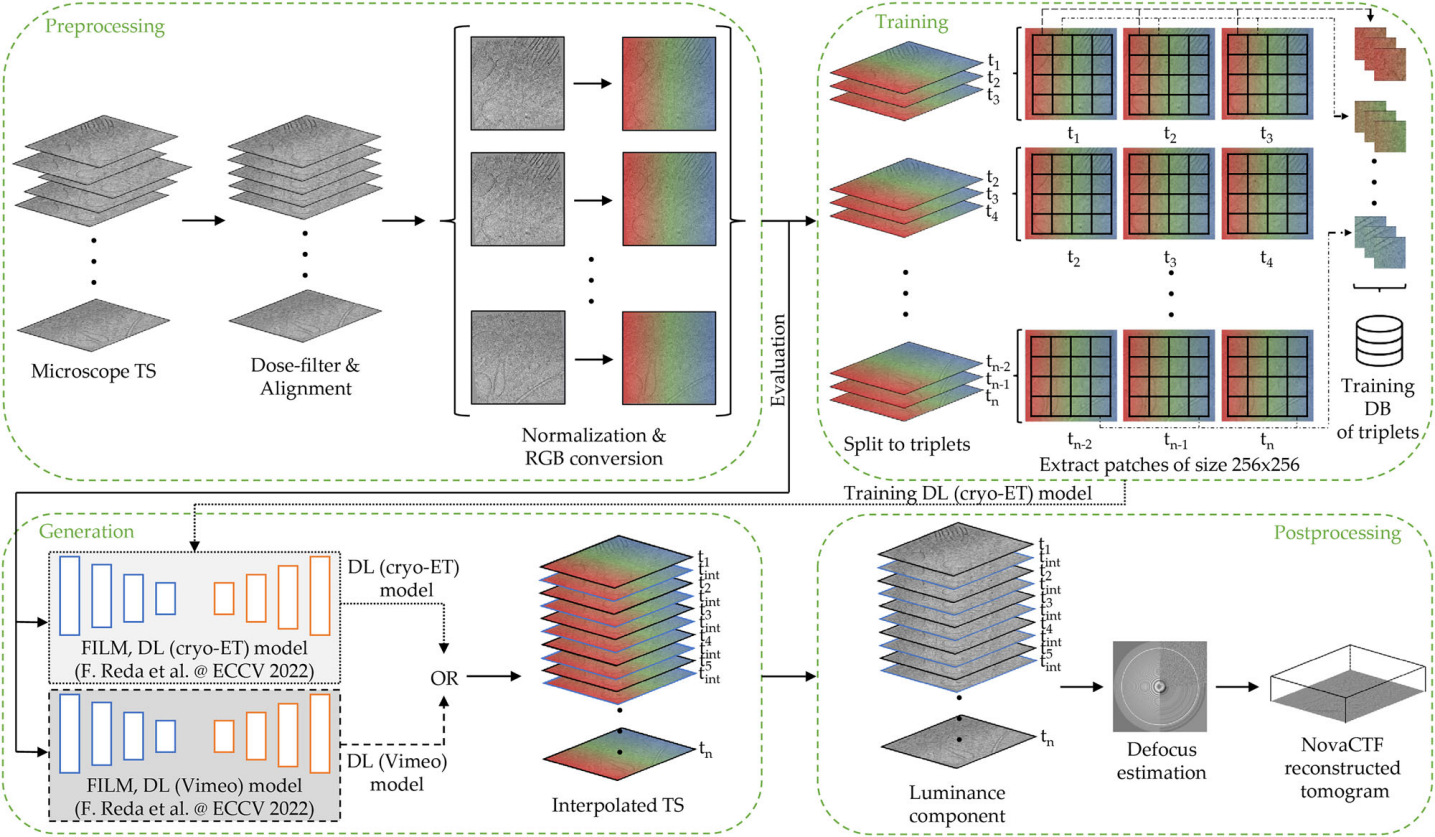
The pipeline of cryoTIGER. It consists of preprocessing, training, generation, and postprocessing steps and incorporates a deep learning frame interpolation model into cryo-ET reconstructions. Individual steps are described in detail in the main text.

FILM employs a multi-scale feature extractor^35^ that shares weights across the scales and presents a “scale-agnostic” bidirectional motion estimation module. This approach relies on the notion that large motion at finer scales should be similar to small motion at coarser scales, thus increasing the number of pixels available for large motion supervision. Similar to how the module ensures consistent motion representation across varying scales, this principle can be applied to tilted images, where key features must remain recognizable despite geometric distortions. By maintaining adaptability and efficiency across transformations, the method aligns well with the challenges of our setup, ensuring robust performance under varying tilt angles. In order to accommodate the FILM framework for cryo-ET data, we made multiple adjustments, as shown in Fig. 1.

Our tilt-series processing pipeline consists of *preprocessing*, *training*, *generation*, and *postprocessing* steps. In *preprocessing*, raw microscope tilt series images undergo the basic operations of dose filtering, alignment, normalization, and conversion from grayscale images to colored images with red, green, and blue (RGB) channels in order to make them compatible with the FILM algorithm. During the *training* step, we utilized data from multiple cryo-ET datasets, see Table 1. The training data were collected with different pixel sizes and tilt increments to increase the robustness of the trained DL models. The majority of these tilt images were acquired with a more commonly used increment of two or three degrees, but we included a one-degree tilt increment dataset as well, in order to have items with smaller motion in the training dataset.

**Table 1:** Summary of training tilt series

Sample	Pixel size	Tilt increment	# of TS
*Dictyostelium discoideum*	1.971	1	49
*Dictyostelium discoideum*	1.971	2	77
*Dictyostelium discoideum*	1.971	3	159
Human T cells	1.971	2	32
Human embryonic kidney (HEK Flp-In T-Rex)	1.971	2	10
Human embryonic kidney (HEK-293)	1.188	2	18
Human skin fibroblasts	2.414	2	30
**SUM**			375

Breakdown of the pixel size (in Å), tilt increment (in degrees), and number of tilt series used to train DL (cryo-ET) model. The data were collected independently by five experienced users. Additional information about other tested models and their configurations is in Supplementary Table 1.

Supplementary Table 1 provides a list of models that we considered and trained for this work. When we trained the model on larger tilt increments only, the performance slightly worsened (see Supplementary Fig. 1). From now on, we refer to DL (cryo-ET) as the model trained on 317,312 triplets from 375 tilt series, which is highlighted in Supplementary Fig. 2.

The FILM framework proposed a unified architecture for image interpolation, which is trainable from regular image triplets alone^34^. In our setup, a triplet refers to a set of three consecutive tilt images, where the two external ones are used to interpolate a tilt image between them, and the middle tilt image is used as the ground truth image for comparison. Therefore, in order to train the network with cryo-ET data, we first split tilt images into triplets. Because the input size of triplets for training using the FILM algorithm is 256 × 256, we further divided each tilt into patches of this size and stored them in a training database.

In the *generation* step, we tested two models: a DL (Vimeo) model, trained on samples from the Vimeo-90K dataset^36^, and a DL (cryo-ET) model, trained on cryo-ET data to interpolate additional tilts between the ones acquired physically. During the *postprocessing*, the luminance component representing the overall brightness of an image is extracted (see Methods “Preprocessing” for details), followed by defocus estimation using Gctf^37^, and tomographic reconstruction using novaCTF^38^, which performs correction of contrast transfer function (CTF) in 3D. Note that since we use aligned tilt series for interpolation, the generated images do not require any alignment prior to the reconstruction.

In the following subsections, we focus on demonstrating how DL models outperform naive linear interpolation and no-interpolation scenarios. Our emphasis is on showing that DL-based interpolation consistently enhances performance across all evaluated tasks.

### Analysis of 2D interpolated tilt images

We first analyzed the generated data in 2D by comparing their quality to the available ground truth (GT) images. We used Dictyostelium discoideum tilt series acquired with a one-degree tilt increment (see Table 1) and split them into even and odd tilts. Odd tilts, starting from index one, were used as input to generate interpolated tilts, while even tilts, starting from index two, served as GT data. We investigated three different interpolation approaches and compared them to the GT. The first approach is a linear interpolation, in which we calculate the pixel-wise average between each pair of adjacent tilt images. This method serves as a simple baseline without any deep learning component. We also considered cubic and tricubic interpolation models, but their performance was worse compared to the linear interpolation model (see Supplementary Fig. 3). The remaining two approaches utilize the FILM framework to generate interpolated samples, where we employed either a DL (Vimeo) model trained on samples from the Vimeo-90K dataset^36^ or a DL (cryo-ET) model as described above.

Vimeo-90K comprises 89,800 sequences of seven frames each, significantly exceeding the size of the cryo-ET dataset derived from 375 tilt series. Due to hardware limitations (500 GB RAM), we were unable to scale the training dataset or model further. While the interpolation framework itself is not cryo-ET specific, the unique challenge lies in handling tilt-induced motion, which we address in Methods, section “Preprocessing”

**Fig. 2:**
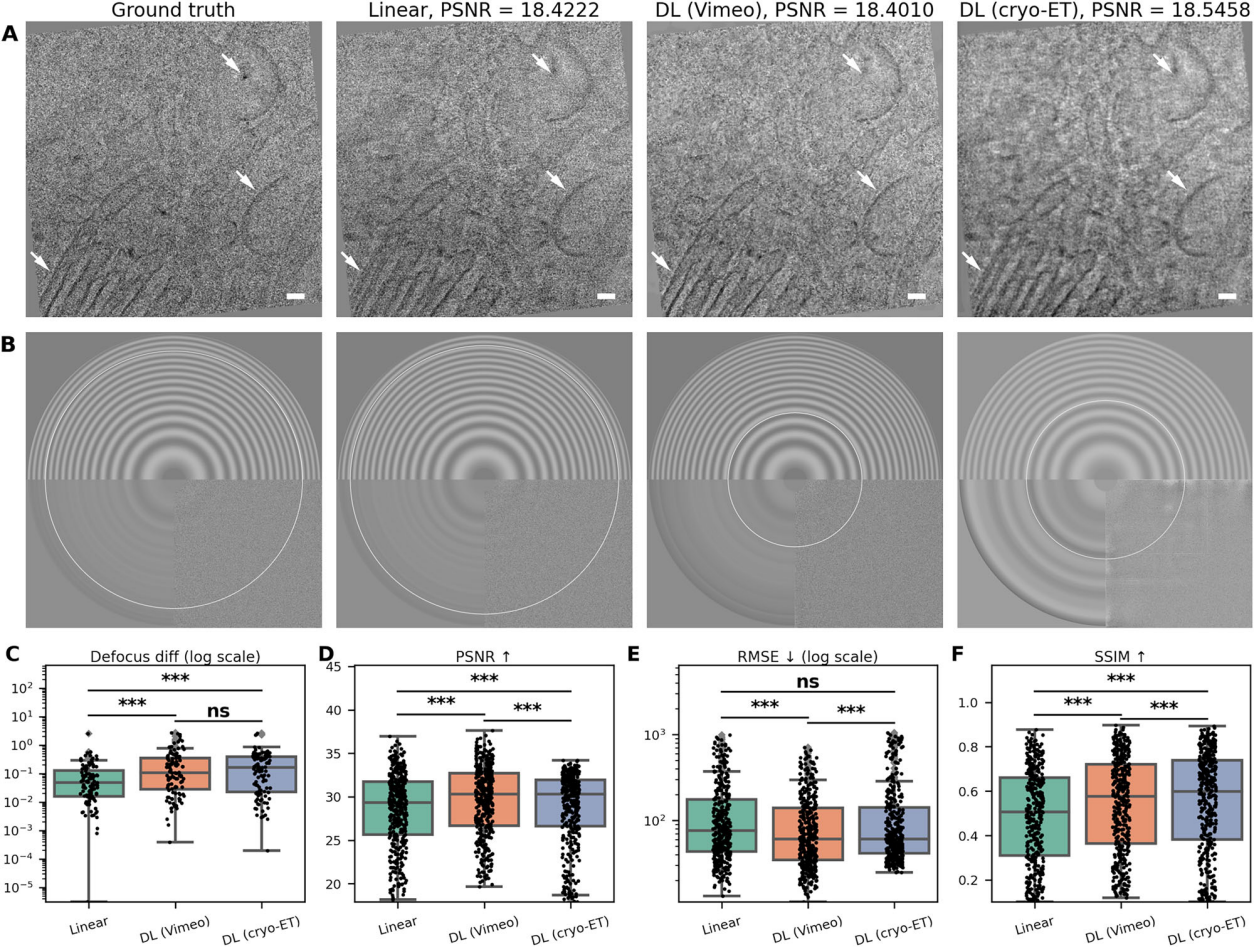
Comparison of 2D tilt series images generated using different interpolation methods. **A** Visual differences between features in the GT image and interpolated images. White arrows indicate structural features with more pronounced differences between approaches. **B** Corresponding defocus estimation from the Gctf diagnostic output. The white circle denotes the resolution up to which the CTF was reliably fitted. **C** Difference in defocus values (µm), *n* = 111. **D** Peak signal-to-noise ratio (PSNR), where higher values indicate better quality (measured in decibels), *n* = 367. **E** Root mean square error (RMSE), where lower values indicate better performance, *n* = 367. **F** Structural similarity index (SSIM), where higher values indicate greater visual similarity to the original image, *n* = 367. Pairwise group comparisons were assessed using the paired samples *t*-test. Significance is indicated as *** for *p* < 0.001, ** for *p* < 0.01, * for *p* < 0.05, and “ns” for non-significant. Boxplots show medians, interquartile ranges (IQR), and whiskers up to 1.5× IQR; outliers and all individual values are overlaid as points. Scale bar: 50 nm. Extended information is in Supplementary Table 3.

Figure 2 presents a comparison of interpolation methods against ground truth data. It is important to note that the dose distribution in our evaluation is to some extent artificial, as discussed in detail later in the text. We first examined visual accuracy, where all tested methods generated realistic outputs (panel A). The DL (cryo-ET) model produced slightly more blurred tilts but with higher contrast compared to the other two methods. Regarding CTF estimation, the linear interpolated image showed the most resemblance to the GT, while the DL (Vimeo) model and the DL (cryo-ET) model exhibit lower fitting accuracy (panel B). The DL (cryo-ET) model exhibits more artifacts in Fourier space, which is caused by noise present in the microscopic images used for the training (see Supplementary Figs. 4–6). Generally, the defocus values of the images computed by the linear interpolation are closer to those of the GT (panel C), with the median defocus difference being around 50 nm, while for the DL models, the median is 100 nm for DL (Vimeo) and 160 nm for DL (cryo-ET).

For assessing visual similarity quantitatively, we employed three metrics. Firstly, the peak signal-to- noise ratio (PSNR), which measures the ratio of maximum signal power to noise power. Secondly, the root mean square error (RMSE), which calculates the square root of the average squared differences between predicted and actual values, weighting larger errors more heavily. Lastly, the structural similarity index (SSIM), which evaluates perceived image quality by comparing luminance, contrast, and structural information, with values ranging from −1 (inverse structure) to 1 (identical images). See Methods “2D image comparison metrics” and Supplementary Information for mathematical definitions of all three metrics and descriptions of their units. Fig. 2D–F shows comparisons between GT tilts and interpolated tilts using PSNR, RMSE, and SSIM, respectively. For PSNR, DL-based models achieve values closer to GT compared to linear interpolation, which suggests that the generated tilt series are more similar to the GT tilt images. The RMSE analysis indicates that the DL (cryo-ET) model exhibits a higher prevalence of outliers compared to the DL (Vimeo) model. However, the SSIM evaluation demonstrates a slight performance improvement in structural similarity for the DL (cryo-ET) model in comparison to the DL (Vimeo) model. Although none of the presented interpolation methods was superior, the results overall highlight that we can faithfully simulate microscope tilt images using any of them.

### Template matching on tilt-series with interpolated tilts

To evaluate the performance of the proposed approach in generating realistic intermediate tilt images, we integrated generated images into our reconstruction pipeline (Fig. 1). To quantify the impact of the interpolation for particle identification, we used high-confidence 3D template matching (TM)^15^ as implemented in GAPSTOP^TM^ ^14,39^. We used a subset of 20 tilt series from the dataset EMPIAR-12454^40^ and applied TM to localize nuclear ring subunits of the nuclear pore complex (NPC NR).

As a ground truth, we used the manually curated list of particle positions provided by the authors^40^. We further used a subset of 24 tilt series from dataset EMPIAR-11899^41^ to demonstrate the performance on the 80S ribosome using the particle positions provided by the authors of the original study. We computed the F1 score, precision, recall, and the area under the precision-recall curves (PR-AUC) to assess the performance of TM using different interpolation types (see Methods “Metrics for evaluating peak selection” and Supplementary Information for more details and definitions).

The evaluation was conducted under two experimental scenarios. In the first scenario, we started with a tilt series acquired with a tilt increment of two degrees. We then removed every second tilt, resulting in a reduced tilt series with a tilt increment of four degrees, which served as a baseline. Subsequently, we interpolated one tilt between each pair of remaining tilts, thereby constructing a tilt series with an increment of two degrees. We also evaluated interpolation of more than one tilt, but the performance typically decreased with additional interpolated tilts (see Supplementary Fig. 7).

This setup allowed us to compare three distinct conditions: (1) the baseline tilt series with missing tilts after the removal step, (2) the interpolated tilt series where missing tilts were replaced with interpolated samples to restore the original number of tilts, and (3) the GT tilt series, acquired directly from the microscope, which contained all the original tilts prior to the artificial removal step (see Supplementary Fig. 8 for power spectra depiction for all three cases). By comparing these conditions, we assessed the extent to which the interpolation improved tomogram properties and subsequent downstream analyses relative to the baseline without interpolation. Additionally, we compared the results to the GT reconstruction.

**Fig. 3:**
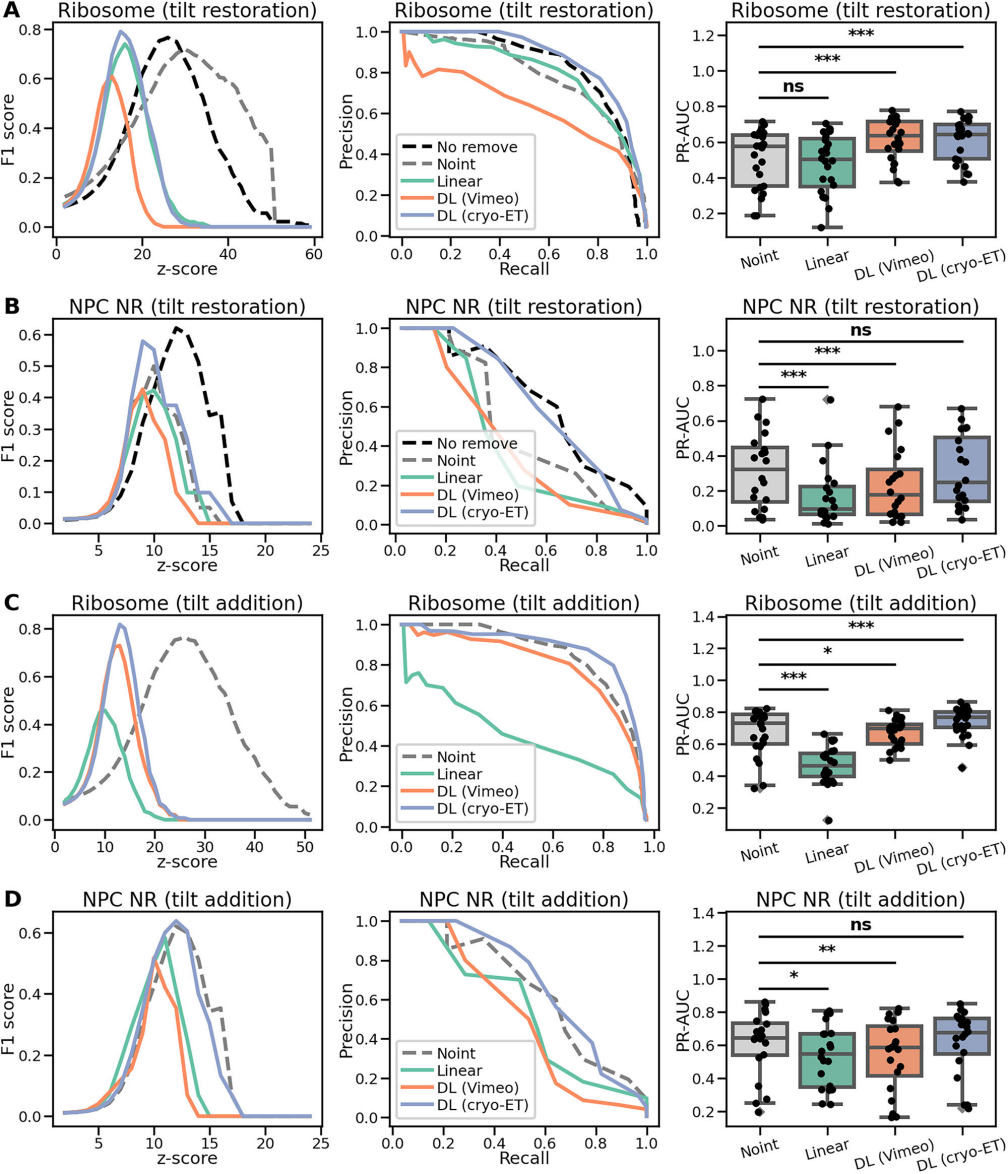
Comparison of template matching results for restoring and adding tilt images using interpolation. **A** Results for restoring removed tilt images in the 80S ribosome dataset. F1 scores (left) and precision-recall curves (middle) are shown for a single representative tomogram. The area under the precision-recall curve (PR-AUC) was calculated across all tomograms in the dataset (right), *n* = 24. **B** Same as (**A**), but for tomograms containing NPC NR, *n* = 20. **C** Results for adding new interpolated tilt images in the 80S ribosomes dataset, *n* = 24. **D** Same as (**C**), but for tomograms containing NPC NR, *n* = 20. Pairwise group comparisons were assessed using the Wilcoxon signed-rank test (two-sided). Significance is indicated as *** for *p* < 0.001, ** for *p* < 0.01, * for *p* < 0.05, and “ns” for non-significant. Boxplots show medians, interquartile ranges (IQR), and whiskers up to 1.5× IQR; outliers and all individual values are overlaid as points. The comparison results for all the pairs are summarized in Supplementary Table 4.

The results for restoring removed tilts are presented in Fig. 3A for the 80S ribosome and in Fig. 3B for NPC NR. F1 scores and precision-recall graphs are illustrated on a single representative tomogram, while the PR-AUC values were extracted for all tomograms from each dataset, offering a broader and more representative overview of the model performance with statistical tests.

It is important to note that the removal of tilts introduced a dose distribution that is, to some degree, artificial. In the case of acquiring data with a 4-degree tilt increment, one would either keep a lower total dose, retaining more high-resolution content, or one would use more dose per image to achieve better contrast. In our case, the total dose is not reduced, and the retained tilt images have low SNR, thereby making the interpolation task more challenging. Nevertheless, we consider this approach to be the closest approximation to a direct comparison between reconstructions with and without interpolation data.

In the second testing scenario, additional interpolated tilts were added to a full experimental dataset (without removed tilts), and the same set of comparisons was performed. Note that in this case, the non-interpolated data contains fewer images in the tilt series because we interpolated tilts that were not acquired. However, this test accounts for a more realistic experimental electron dose. The results are presented in Fig. 3C, D in the same order as for the first testing scenario.

To better assess the significance of our results, we performed pairwise group comparisons and used the Wilcoxon signed-rank test (two-sided) to evaluate them (see Fig. 3 and Supplementary Table 4). Linear interpolation performed the worst in all tested cases. Notably, in the tilt restoration test for NPC NR and the tilt addition test for 80S ribosome, it significantly degraded the signal content, even when it was compared to the non-interpolated data. This is especially surprising considering Fig. 2B, where linear interpolation had the best CTF fitting.

In the case of the DL (Vimeo) model, differences relative to the non-interpolated data were less pronounced. Nevertheless, this model outperformed the non-interpolated baseline in tilt image restoration for the 80S ribosome dataset. By contrast, the DL (cryo-ET) model consistently outperformed all other evaluated methods, including the non-interpolated variant. While the improvements were not statistically significant for the NPC NR dataset in the overall analysis, a more detailed evaluation revealed notable benefits. Specifically, when analyzing cross-correlation (CC) values at the ground truth particle positions (see Supplementary Fig. 9), the DL (cryo-ET) model showed a significant increase in measured CC values. Pairwise comparisons of CC values at each ground truth position further supported these findings (see Supplementary Figs. 10, 11 for NPC NRs and 80S ribosomes, respectively), where the improvements achieved by DL (cryo-ET) interpolation model were more pronounced and statistically significant.

To complement the PR-AUC plots presented in Fig. 3, we provide a detailed pairwise comparison of AUC values across all methods. These results are shown in Supplementary Fig. 12 for 80S ribosome tilt restoration, Supplementary Fig. 13 for NPC NR tilt restoration, Supplementary Fig. 14 for 80S ribosome tilt addition, and Supplementary Fig. 15 for NPC NR tilt addition.

These comprehensive comparisons further reinforce the consistent advantage of the DL models, especially the DL (cryo-ET) model, which outperformed the non-interpolated baseline across all evaluated conditions. The results highlight the robustness and effectiveness of our approach for enhancing particle localization in cryo-ET data.

### Improving the 3D structure of nucleosomes using cryoTIGER

**Fig. 4:**
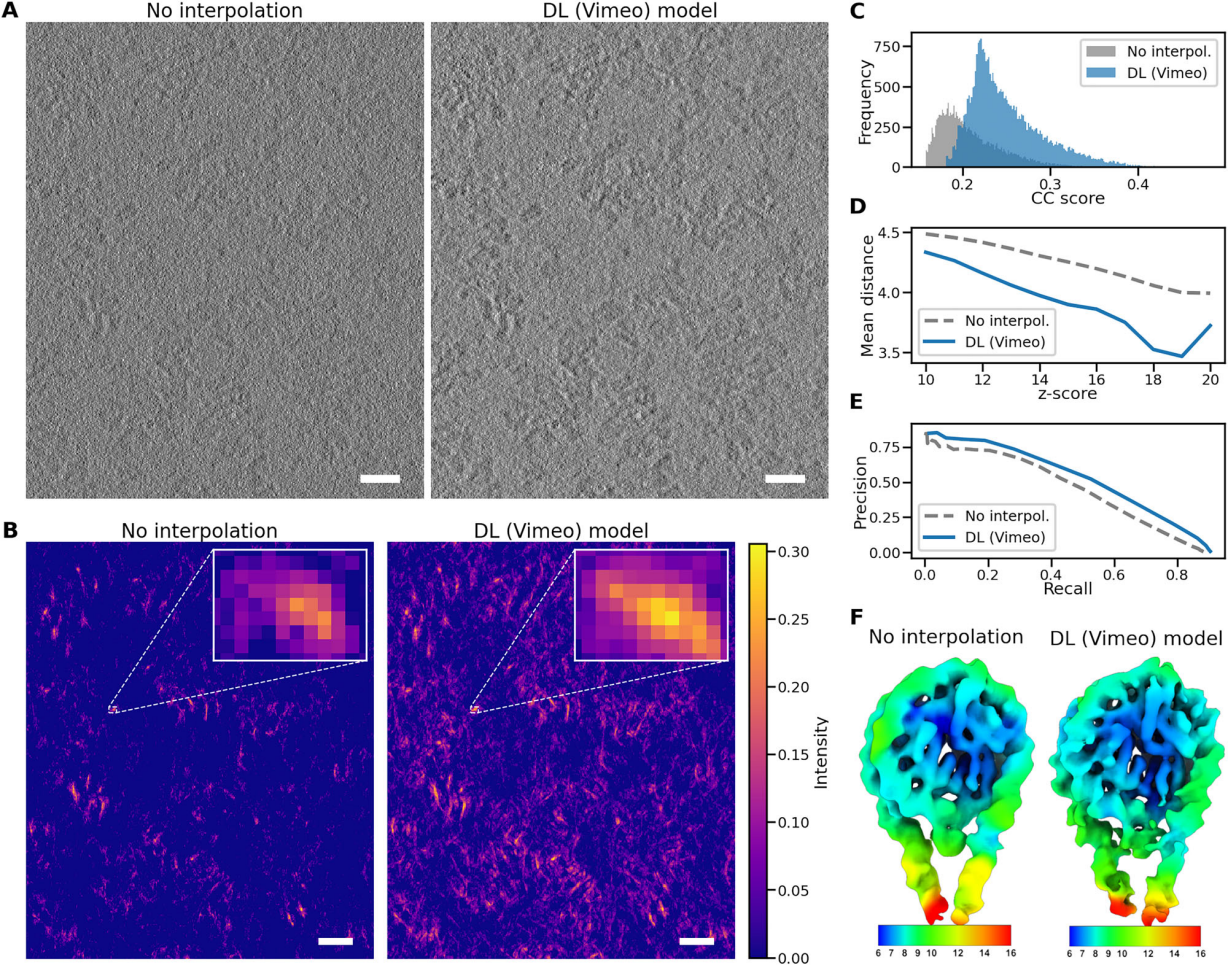
Enhanced particle picking and 3D structure determination. **A** 2D slice from the tomogram reconstructed without interpolated tilt images (left) and with images interpolated using the DL (Vimeo) model (right), where the contrast for improved after adding interpolated tilts. **B** The same slice after the template matching for the tomogram in panel (**A**) (slightly rescaled due to the intensity scale bar at the right), shows markedly improved cross-correlation peaks for the interpolated data (right). **C** Histogram of CC scores from selected peaks, illustrating an increase in both absolute values and frequency for the interpolated data. **D** Average distance of CC peaks to the refined positions found during STA, showing improved localization precision for the interpolated data. The mean distance is measured in voxels with a voxel size of 3.942. **E** Precision-recall curves, with a higher PR-AUC value for the interpolated data (0.4769) compared to non-interpolated data (0.4148). **F** 3D structures calculated based on non-interpolated (left) and interpolated positions, colored by local resolution (color bar in Å). Although the structure based on interpolated positions has a similar resolution, it reveals more detailed structural features. Scale bar: 20 nm.

Encouraged by these results, we next applied DL interpolation models to enhance the localization of nucleosomes. Note that the full analysis of nucleosomes is the main focus of a separate study^42^, and here we present only improvements introduced by our framework. Nucleosomes have a molecular weight of approximately 250 kDa, which, together with the crowded environment present in the nucleus and the lack of visual validation of their position, makes them an especially challenging target to identify in tomographic data. We analyzed a dataset of 14 tomograms acquired from the nuclear periphery of T cells with a tilt step of two degrees. We employed our framework to interpolate additional tilt images in between the experimental ones, resulting in a tilt series with a tilt step of one degree using the DL (Vimeo) model. The use of the interpolated tilt series led to improved contrast in tomograms in comparison to those created from non-interpolated tilt series. As a result, individual features became more visible, as can be seen in Fig. 4A, showing 2D slices from the reconstructed tomograms. The presented tomograms were reconstructed using novaCTF^38^ with no additional postprocessing. The improved contrast is also confirmed by Standard Deviation Contrast (non-interpolated data 0.0109 and interpolated data 0.0217) which quantifies how much the pixel values deviate from the average intensity across the entire image and by Gradient-Based Contrast (0.0034 and 0.0061, respectively) that measures the intensity variation by looking at how pixel values change between neighboring pixels (see Methods “Contrast-based metrics for visual quality” for more details).

After running 3D template matching in GAPSTOP^TM^ ^14^ on these tomograms, more distinct cross-correlation peaks with generally higher values were obtained. This can be observed in Fig. 4B, showing 2D slices from the TM outcome. Looking closer to the CC peaks reveals not only lower CC values for the non-interpolated data but also a smaller cluster of distinct CC values around the peak, as can be seen in the insets of Fig. 4B. The quantification of the CC values across the whole dataset is depicted in Fig. 4C. Since there was no GT for nucleosome positions, it was necessary to validate that the TM positions with the highest CC scores correspond to actual nucleosome locations in the tomograms. The authors^42^ validated the nucleosomes’ positions by manually creating a binary mask to separate the nucleus from the non-nucleosome-containing cytoplasm. Subsequently, only the nuclear peaks that were thresholded by the 99th percentile of the cytoplasmic peaks were considered. The baseline for both the non-interpolated and interpolated versions was established in this manner^42^.

Overall, in the 14 tomograms, we detected ∼18k nucleosome particle positions in the non-interpolated condition, while the interpolated variant identified ∼33k positions. We want to emphasize that we used interpolated data to detect positions, but the actual particles for STA were extracted from the tomograms without interpolation. Interpolated data could potentially contain additional high-resolution details that were not experimentally verified. Therefore, we use them to improve the reconstruction pipeline, but in order to determine the 3D structure, only experimentally acquired data are utilized.

The average distance between the matches in non-interpolated and interpolated peak positions confirmed that the latter are closer to the positions obtained after STA refinement and hence more precise in terms of their localization (Fig. 4D). We computed the precision-recall curves for both tested conditions (Fig. 4E), where we observed an increase in PR-AUC value from 0.4148 for non-interpolated data to 0.4769 for interpolated data. A higher PR-AUC indicates that the model is better at correctly identifying positive instances without producing a large number of false positives.

When ∼33k nucleosome particle positions from the interpolated variant were used in the STA, it led to marginal improvement in resolution (from 8.4 to 8.3 Å, see Supplementary Fig. 16). However, the map obtained from the interpolated-based particle list has more pronounced structural details as shown in Fig. 4F. The enhanced details are especially visible on the DNA linker arms. This use case shows the great potential of interpolation for reliable localization of small features in crowded cellular environments (an increase in founded positions by 87.33% supported by 14.97% improvement in PR-AUC).

### Refined deep-learning particle picking

The aforementioned use cases primarily focus on downstream analyses using the template matching pipeline. Here, we demonstrate the cryoTIGER application with DeePiCt^17^, an open-source deep-learning framework designed for supervised segmentation and macromolecular complex localization. DeePiCt models, trained on experimental cryo-ET data, are broadly applicable across species and datasets.

To evaluate the impact of interpolation on downstream particle localization, we utilized the Colab notebook provided by the authors of DeePiCt^17^, which allows inference using 3D convolutional neural network models. Specifically, we used the pretrained model available in the Colab notebook, optimized for ribosome localization, and applied it to a set of 24 tomograms. Predictions were performed both on the original (non-interpolated) data and on data processed using three different interpolation methods: linear interpolation, the DL (Vimeo) model, and the DL (cryo-ET) model.

**Fig. 5:**
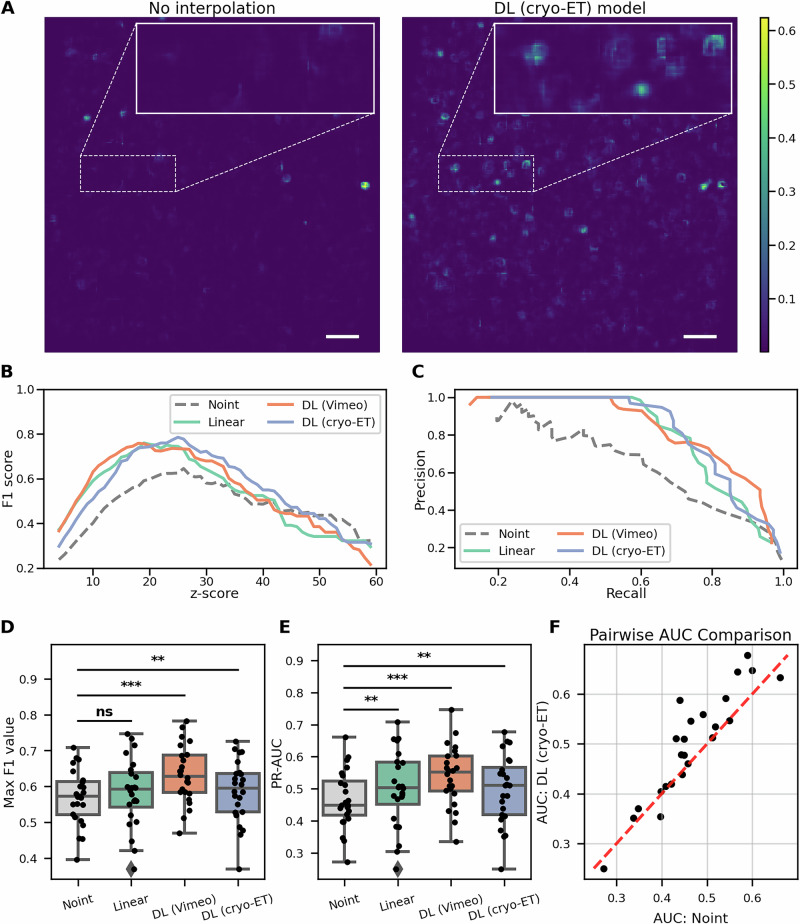
Particle picking of ribosomes with DeePiCt. **A** 2D slice from the probability map generated by DeePiCt using non-interpolated data (left) and interpolated data with the DL (cryo-ET) model (right). The probability scale is identical for both maps. Without interpolation, few peaks are visible, but after adding interpolated frames, many more positions are revealed. **B** F1 score for a representative tomogram, showing improved DeePiCt performance with interpolation compared to the manual ground truth. **C** Precision-recall curve comparison for the same tomogram, demonstrating enhanced DeePiCt performance with interpolation. **D** Maximum F1 scores across 24 tested tomograms (*n* = 24); all the pairs are summarized in Supplementary Table 5. **E** Area under the precision-recall curve for 24 tested tomograms (*n *= 24); all the pairs are summarized in Supplementary Table 5. **F** Pairwise comparison of PR-AUC values between the non-interpolated data and the DL (cryo-ET) model, *n* = 24. Statistical comparisons were assessed using the Wilcoxon signed-rank test (two-sided). Significance is indicated as *** for *p* < 0.001, ** for *p* < 0.01, * for *p* < 0.05, and “ns” for non-significant. Boxplots show medians, interquartile ranges (IQR), and whiskers up to 1.5× IQR; outliers and all individual values are overlaid as points. Scale bar: 40 nm.

As illustrated in Fig. 5A, which presents representative 2D slices from the resulting 3D probability maps, interpolation led to an increased density of high probability peaks compared to the non-interpolated baseline. Among the methods tested, the DL (cryo-ET) model produced the highest probability values, suggesting improved localization performance. The enhanced contrast in the probability maps indicates that the proposed interpolation approach preserves structural features more effectively, thereby facilitating more accurate ribosome detection by DeePiCt.

To validate the predicted particle positions, we compared them against a manually curated ground truth list, as described in the section “Template matching on tilt-series with interpolated tilts”. Evaluation was conducted using the F1 score and precision-recall curves, as shown in Fig. 5B, C. Across all interpolation strategies, interpolated data consistently yielded higher F1 scores and improved precision-recall characteristics compared to the non-interpolated baseline.

To further support our findings, we computed summary statistics including the maximum F1 score and the area under the precision-recall curve, both of which provide robust, threshold-independent assessments of model performance. As shown in Fig. 5D, E, the use of interpolated tilt images substantially improves localization accuracy across both evaluated deep learning-based methods. The comparison results for all the pairs are summarized in Supplementary Table 5.

In Fig. 5F, we present a pairwise comparison of PR-AUC values between the non-interpolated data and the DL (cryo-ET) model. The results show improvements in 18 out of 24 tomograms, where data points lie above the identity line (red), indicating superior performance of the interpolated variant. Only six tomograms show slightly better results with non-interpolated data; however, the deviations from the identity line in these cases are minimal, suggesting marginal differences.

A complete set of pairwise PR-AUC comparisons for ribosome particle picking using DeePiCt is provided in Supplementary Fig. 17, further demonstrating the consistent advantage of the interpolation approach.

### Enhanced membrane segmentation

We also evaluated the impact of the interpolation on DL-based, fully automated membrane segmentation as implemented in MemBrain v2^43^. A core module of MemBrain v2 employs a 3D-UNet architecture optimized for cryo-ET membrane segmentation, where the provided models were trained on diverse cryo-ET data, resulting in robust and widely-used software for membrane segmentation^44–46^.

To source publicly available annotated data in standardized formats, we utilized the cryo-ET Data Portal^47^. In the portal, we identified four tilt series (128_2, 129_2, 133, 141_3) from the dataset CZCDP-10004, containing all necessary reconstruction files as well as a hybrid segmentation mask to validate the suggested DL interpolation strategy. The hybrid annotation method combines tomogram denoising, 3D-UNet-based membrane segmentation, and manual postprocessing. We deliberately selected this hybrid approach to more accurately compare the performance of fully automated MemBrain v2 on non-interpolated and interpolated datasets.

We ran MemBrain v2 on the four tomograms reconstructed without interpolation, with linear interpolation, and with interpolation based on DL models. We assessed the quality of segmentation using the Jaccard Index, the Dice Coefficient, and the Hausdorff Distance (see details on metrics for evaluating segmentation in Supplementary Information for formulas and more details).

**Fig. 6:**
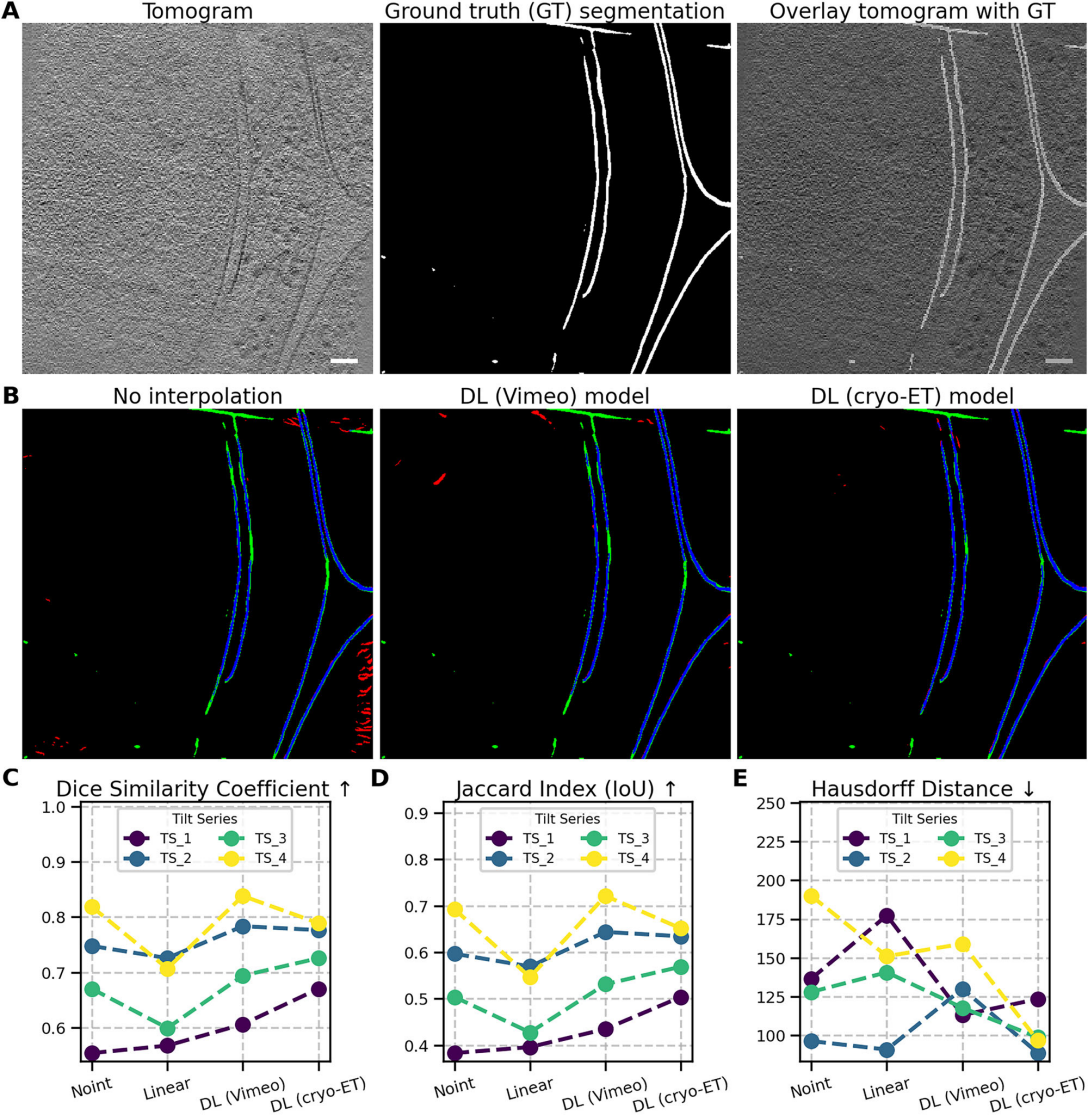
Enhanced membrane segmentation using MemBrain v2. **A** Representative tomogram slice from a tilt series downloaded from the cryo-ET Data Portal^47^ and reconstructed using our pipeline (left). The same slice with the hybrid ground truth segmentation (manually curated) is depicted in white (middle). The tomogram slice overlaid with the GT segmentation (right). **B** Comparison of the non- interpolated version with interpolation using the DL (Vimeo) model and the DL (cryo-ET) model. Green regions represent areas in the GT but not detected by MemBrain v2 (false negatives), red regions were detected by MemBrain v2 but are not in the GT (false positives), and blue regions were detected by both MemBrain v2 and the GT (true positives). **C** Overlap-based dice similarity coefficient for four tested tomograms, ranging from 0 (no overlap) to 1 (full overlap). **D** Overlap-based Jaccard Index for the same tomograms, also ranging from 0 to 1. **E** Hausdorff distance measuring how far the tested segmentation outlines are from the GT segmentation. Scale bar: 60 nm.

Across all three metrics, we observed a consistent improvement in the outputs of MemBrain v2 when using the DL interpolation workflow (see Fig. 6). After adding interpolated frames generated by DL models, some false positive segmentation artifacts were removed (indicated in red in panel B), and the automated segmentation more closely matched the ground truth hybrid annotation, with fewer false negatives (indicated in green in panel B). These results strongly demonstrate the potential of interpolation to enhance fully automated membrane segmentation.

Additionally, we observed a clear increase in the Dice Coefficient (panel C) and Jaccard Index (panel D), along with a decrease in Hausdorff distance (panel E), further confirming the improvement in segmentation accuracy due to more precise reconstruction enabled by the inclusion of interpolated frames.

### Enhanced microtubule segmentation

Lastly, we evaluated the impact of interpolation on fully automated microtubule (MT) segmentation using DeePiCt. This extends the framework to encompass both membrane segmentation with MemBrain v2 and microtubule segmentation with DeePiCt, which has also been used for ribosome particle picking. These applications highlight the conceptual and methodological parallels between particle picking and segmentation in cryo-ET analysis.

**Fig. 7:**
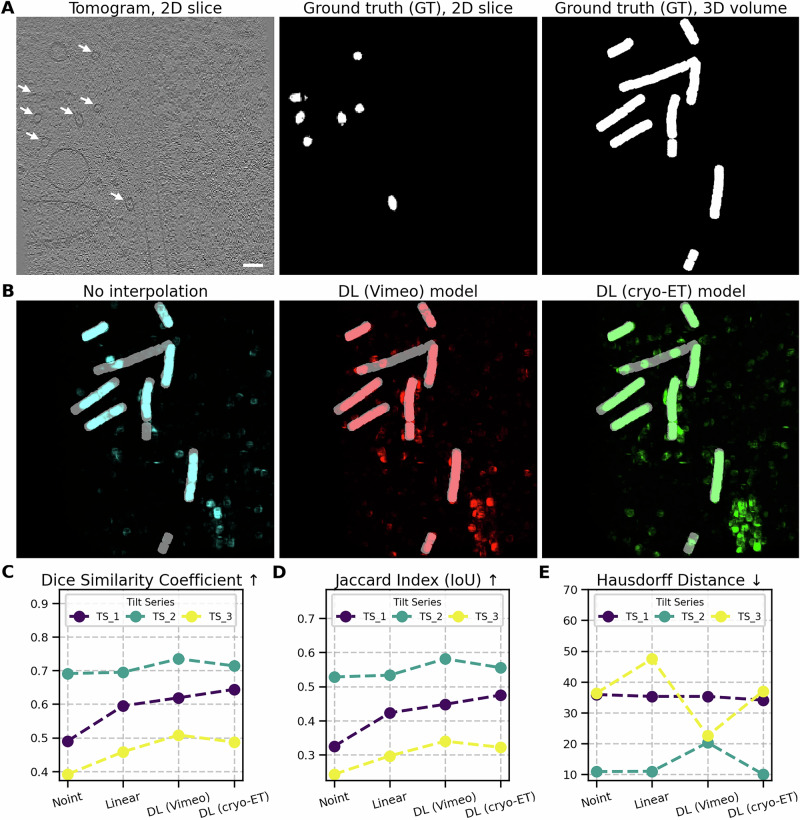
Enhanced microtubule segmentation using DeePiCt. **A** Representative tomogram slice with arrows indicating the positions of microtubules (left). The corresponding ground truth segmentation is depicted in white (middle), alongside a 2D projection of the GT segmented 3D volume for visualization (right). **B** Comparison of the non-interpolated version (in cyan) with interpolation using the DL (Vimeo) model (in red) and the DL (cryo-ET) model (in green). **C** Overlap-based dice similarity coefficient for three tested tomograms, ranging from 0 (no overlap) to 1 (full overlap). **D** Overlap-based Jaccard Index for the same tomograms, also ranging from 0 to 1. **E** Hausdorff distance measuring how far the tested segmentation outlines are from the GT segmentation. Scale bar: 60 nm.

Details on the generation of ground truth (GT) data for microtubule segmentation are provided in Methods, section “Ground truth data for microtubule segmentation”. For evaluation, we segmented tomograms under all tested conditions using DeePiCt, which includes a dedicated model for MT segmentation. The results are summarized in Fig. 7. Panel A displays a representative 2D slice from one tomogram, alongside a corresponding 2D slice and a 2D projection of the GT segmentation from the full 3D volume. A comparison is shown between the non-interpolated version (cyan), interpolation using the DL (Vimeo) model (red), and interpolation using the DL (cryo-ET) model (green). The deep learning-based models produced more complete segmentations, especially near microtubule ends, which are often truncated in the non-interpolated condition.

As with membrane segmentation, we assessed segmentation quality using the Jaccard Index, Dice Coefficient, and Hausdorff Distance (see Supplementary Information for metric definitions and formulas). We observed a similar trend: an increase in Dice Coefficient (panel C) and Jaccard Index (panel D), along with a decrease in Hausdorff Distance (panel E), indicating improved segmentation accuracy due to interpolation.

## Discussion

To address the challenge of angular spacing in tomographic reconstructions, our study introduces a specific application of an existing frame interpolation framework, tailored to the cryo-ET pipeline. By interpolating images between acquired tilts, our approach effectively increases angular sampling, enhancing the signal content in the reconstructions. To ensure robustness, we developed a custom model through extensive training on diverse datasets, varying in both cellular content and acquisition setup. Validation experiments demonstrated that DL-based interpolation generates images that enhance tomogram reconstruction and outperform conventional linear interpolation.

The impact of interpolated images on tomogram properties was comprehensively evaluated using template matching, DL-based particle localization and segmentation with DeePiCt, and DL-based segmentation using MemBrain v2 across diverse datasets and targets. The results for DL-based interpolation consistently outperformed those obtained from linear interpolation, with notable improvements observed in all cases. This was surprising given the generally good performance of linear interpolation in 2D. One possible explanation is that DL-based approaches better preserve structural content (see Fig. 2F) by considering contextual information from the surrounding area, whereas linear interpolation operates on a pixel-by-pixel basis, making it less suitable for noisy or imperfectly aligned data. This could also explain why, in some cases, we observed that linear interpolation actually worsened the results of downstream processing. We hypothesize that these cases correspond to tilt series with poorer alignment quality, likely due to higher noise levels.

For the comparison of the DL-based models, while the DL (cryo-ET) model often excelled, there were instances where the DL (Vimeo) model performed better or similarly well. Given the diametrically different images used for the training, such results might seem counterintuitive, as one would assume that training using domain-specific data should improve the model and its performance. However, the extent of improvement in DL methods depends heavily on both the size and quality of the training datasets. Ideally, one would acquire datasets specifically for training purposes, for example, such as tilt series with 1° angular increments while maintaining the same dose per tilt image as in standard 2° acquisitions. However, such a setup would double the number of tilt images and require halving the angular range (e.g., to ±30°) to keep the total dose constant. This would limit the utility of those datasets exclusively to training.

Furthermore, the number of tilt series required to sufficiently train a deep model would likely exceed the dataset sizes typical of cryo-ET projects. For this reason, we relied on existing datasets, some with 1° angular increments but lower dose, and others with 2° increments and higher dose (where training pairs are spaced 4° apart). We believe that the limited availability of ideal training data, combined with lower computational and memory requirements, is the primary reason why the model trained on cryo-ET images showed only modest improvement. On the other hand, this also highlights the robustness of the DL (Vimeo) model: retraining on domain-specific data risks overfitting, whereas the strong performance of the DL (Vimeo) model out of the box suggests good generalization across diverse datasets and imaging conditions.

The most notable advancements were achieved in challenging targets, such as the nucleosome dataset, where our interpolation framework more than doubled the number of reliably localized particles in comparison to the non-interpolated data. Additionally, the automated membrane segmentation results showed greater agreement with manual annotations, highlighting the potential for reduced manual curation. This has far-reaching implications as accurate segmentation of membranes and the localization of associated proteins are essential for advancing cryo-ET studies that link membrane architecture and molecular organization to cellular function. Furthermore, if the automated segmentation is reliable enough, it could replace manual annotations of surfaces needed for surface-based particle localization of pleomorphic assemblies for subtomogram averaging.

The interpolation workflow may influence cryo-ET data acquisition parameters. It enables the use of larger tilt increments with increased electron dose per image without compromising the achievable content. This can improve the performance of downstream processing due to increased SNR. Moreover, for samples that are unusually sensitive to electron dose, an adjusted tilt-acquisition scheme can be combined with tilt interpolation, so data can still be acquired with a reasonable tilt range and sufficient electron dose per image.

Although our study demonstrates the potential of interpolation approaches, it is not without limitations. While interpolated tilts can reduce small gaps in angular sampling, they do not resolve structural data loss associated with missing data across a larger angular space. Attempts to generate more than one interpolated image between two experimental tilt images using the current architecture occasionally resulted in artifacts, particularly when the interpolated tilt images deviated from realistic structural representations (see Supplementary Fig. 1). This highlights the need for careful optimization and evaluation of interpolation outputs to avoid introducing misleading features into reconstructions.

Future work should explore alternative neural network architectures beyond the FILM algorithm to further optimize performance and address existing limitations. For instance, networks designed specifically for extrapolation may hold promise for mitigating the effects of the missing wedge. However, this poses a significant challenge, as it requires the development of approaches that can generalize well without overfitting, especially in the absence of ground-truth data for validation. The integration of extrapolation networks or hybrid models capable of interpolating and extrapolating tilt series data could potentially open new ways for addressing this longstanding issue in cryo-ET.

In conclusion, our study underlines the importance of filling in the angular space between the tilt images and provides a unique computational solution for this problem. Intuitively, one can draw a parallel between interpolation and downsampling. In cryo-ET, tomogram analysis is often performed on binned data, not only because of the large size of tomograms, which makes tasks such as segmentation or particle picking less feasible, but primarily because binning increases the density of low-resolution signal content. This improves contrast, but at the cost of losing high-resolution information, which can be problematic for tasks that rely on both. For example, particle picking of small complexes (such as nucleosomes) requires enhanced contrast while still preserving the ability to distinguish fine structural features. Interpolation increases the low-resolution signal content by densifying the angular sampling, while preserving the original high-resolution data.

The DL-based interpolation approach has shown promising results, enhancing tomogram properties relevant for both particle and feature localization. To facilitate further research and community adoption, we provide our approach as an open framework, cryoTIGER, complete with trained models, laying a solid foundation for the continued exploration of interpolation techniques. By addressing current limitations and pursuing innovative methods, we anticipate further advancements in the tilt-interpolation methods that will continue to enhance cryo-ET reconstructions and facilitate their analyses, thereby advancing structural biology studies.

## Methods

### Preprocessing

The input in our preprocessing pipeline is dose-filtered and aligned tilt series (for the detailed workflow from the raw tilt series to the aligned ones, we refer the reader, for example, to the studies on 80S ribosome^41^ and NPC NR^40^). To accommodate memory constraints, the input tilt series is binned by a factor of 2.

Linear interpolation is performed by computing a pixel-wise average between each pair of adjacent tilt images. For completeness, we also considered the effect of tilting, where the pitch angle is determined as in ref. ^1^. For tilt steps below 3°, the pitch between two neighboring images is below 1, making linear interpolation between corresponding pixels valid. For tilt steps of 4°, the pitch is ~1.2. In this case, one could consider computing linear interpolation between pixels with an offset of 1 or using cubic interpolation, which takes neighboring pixels into account. However, the results shown in Supplementary Fig. 3 suggest that linear interpolation without an offset was superior even for 4° tilt increments. This is most likely due to the downsampling of the data, which combines values from neighboring pixels, thereby diminishing the effects of the pitch. In our study, we only evaluated data with tilt increments below 4°, so this pitch was not considered for linear interpolation.

In the deep learning-based interpolation process, the network requires input data with three color channels. Therefore, the grayscale data were normalized to the 0–255 range, copied into all three channels, and saved in the PNG format.

After executing the interpolation process, the output is generated in the RGB format. Converting this output into a grayscale image using the luminance channel involves combining the three color channels into a single intensity channel that represents the perceived brightness of each pixel. The formula for converting an RGB value to grayscale using the luminance channel is:

$$Y=0.299\,\cdot \,R+0.587\,\cdot \,G+0.114\,\cdot \,B$$

Finally, the image stack is reconstructed using the NumPy library and written out using cryoCAT^48^.

### 2D image comparison metrics

We assessed image similarity using PSNR, RMSE, and SSIM (formulas and more details are in the Supplementary Information). PSNR and RMSE quantify pixel-wise differences, with higher PSNR and lower RMSE indicating better similarity. SSIM measures structural similarity, accounting for luminance, contrast, and texture; values closer to 1 indicate higher similarity.

### Metrics for evaluating peak selection

To evaluate particle selection, we computed precision, recall, F1 score, and PR-AUC (formulas and more details are in the Supplementary Information). A tolerance distance was applied to match detected peaks to baseline positions. Higher values of F1 and PR-AUC reflect better detection performance.

### Contrast-based metrics for visual quality

Standard deviation contrast is a measure used to quantify the contrast of an image by evaluating the variation in pixel intensities. It reflects how much the pixel values deviate from the mean intensity. A higher standard deviation indicates greater contrast, while a lower value suggests lower contrast.

The standard deviation contrast is calculated as the standard deviation of the pixel intensities, given by:

$${{\mbox{Standard}}}\, {{\mbox{Deviation}}}\, {{\mbox{Contrast}}} = {{\rm{\sigma }}} = \sqrt{\frac{1}{m\cdot n}{\sum }_{i=1}^{m}{\sum }_{j=1}^{n}{\left(I\left(i,j\right)-{{\rm{\mu }}}\right)}^{2}}$$

where *I(i,j)* is the pixel intensity at position *(i,j)* and *μ* is the mean intensity of the image, defined as:

$${{\rm{\mu }}}=\frac{1}{m\cdot n}{\sum }_{i=1}^{m}{\sum }_{j=1}^{n}I\left(i,j\right)$$

where *m* and *n* are the height and width of the image, respectively.

A higher standard deviation of pixel intensities indicates higher contrast in the image, whereas a lower standard deviation suggests the image has less contrast.

Gradient-based contrast is a measure used to quantify the contrast of an image based on the spatial variations in intensity between neighboring pixels. This metric highlights areas with sharp intensity changes, such as edges, and is useful for evaluating the sharpness and detail of an image. It is calculated by summing the gradient magnitudes of the image in the horizontal (*x*) and vertical (*y*) directions. The formula is given by:

$${{\mbox{Gradient}}} - {{\mbox{Based}}}\, {{\mbox{Contrast}}} = \frac{1}{m\cdot n}{\sum }_{i=1}^{m}{\sum }_{j=1}^{n}\sqrt{{\left(\frac{\partial I\left(i,j\right)}{\partial x}\right)}^{2}+{\left(\frac{\partial I\left(i,j\right)}{\partial y}\right)}^{2}}$$

where $(\frac{\partial {{\rm{I}}}\left({{\rm{i}}},{{\rm{j}}}\right)}{\partial {{\rm{x}}}})$ is the partial derivative of the image with respect to the horizontal direction *x*, $(\frac{\partial {{\rm{I}}}\left({{\rm{i}}},{{\rm{j}}}\right)}{\partial {{\rm{y}}}})$ is the partial derivative of the image with respect to the vertical direction *y*, and *m, n* are the height and width of the image, respectively.

The partial derivatives are often approximated using convolution filters, such as the Sobel filter^29^, to calculate the gradient in each direction. A higher gradient contrast value indicates a higher degree of contrast in the image, with sharper transitions between pixel intensities.

### Experimental data

#### Parameter setups for TM

In our TM configuration, we used the NPC NR template (EMD-51628)^40^ with a low-pass filter of 23 pixels (corresponding to 30 Å) and angular sampling of 10 degrees on 4× binned data. For detecting ribosomes, we used an 80S ribosome template (EMD-15807)^41^ with a low-pass filter of 30 pixels (corresponding to 23 Å) and angular sampling of 10 degrees on 4× binned data.

#### Nucleosome template matching and subtomogram averaging

The nucleosome structures shown in Fig. 4 were obtained from 14 tomograms of resting T cells. The dataset is the main focus of a separate study^42^, and we therefore refer the reader to the original work for details on the data acquisition parameters and processing. Here we briefly summarize information relevant to our study. The tilt series were acquired with a tilt step of 2° and angular range ±60°, resulting in 61 images and a total electron dose of 135 e^−^ per Å. The pixel size of unbinned data were 1.971 Å.

To compare the performance of the cryoTIGER workflow for TM of nucleosomes, we used GAPSTOP^TM^ ^14^ on novaCTF^38^ reconstructed tomograms that contained either non-interpolated data or data interpolated with the DL (Vimeo) model. The TM was performed on data downsampled by a factor of 2, i.e., pixel size of 3.942 Å.

The nucleosome template was the same for both cases, a lower-resolution in situ nucleosome structure generated from the aforementioned 14 tomograms^42^. The peaks were extracted with the same thresholding approach^42^ and further cleaned by excluding clashing particles with a nucleosome shape mask around each peak in cryoCAT^48^.

The 17,916 (without interpolation) and 33,560 particles (with cryoTIGER workflow) determined through template matching were extracted as unbinned subtomograms in Warp^49^ (for both cases from non-interpolated data) and subjected to subtomogram averaging and alignment in Relion 3.1^50^. The particle set was then imported into M^51^ to perform multi-particle refinement of the tilt series and the final structure. This resulted in a chromatosome structure (containing the core nucleosome with H1 and DNA linkers) resolved to 8.4 Å (with no interpolation) and 8.3 Å (with cryoTIGER workflow) where the latter structure contained more details (Fig. 4G, H).

Note that the chromatosome structure based on the particle list obtained from the interpolated data was further refined and classified, reaching the local resolution of 6.4 Å (7.3 Å overall)^42^. Corresponding Fourier shell correlation (FSC) curves are in Supplementary Fig. 16. This procedure was not reproduced with the particle list based on non-interpolated data due to extensive computational and time costs.

#### Ground truth data for microtubule segmentation

The ground truth segmentation was generated based on positions obtained from TM on three non-interpolated tomograms at 4x binning from the human T cells dataset used also in section “Improving the 3D structure of nucleosomes using cryoTIGER” (see Table 1 and Supplementary Table 2 for more details). The template was generated from EMD-6351^52^ with a low-pass filter of 25px (27 Å). The peaks with a minimum z-score of 9 and a minimum distance of 30 voxels were extracted and manually cleaned for false positives.

### Time complexity and memory limitations

For input tilt series consisting of 61 tilts with the resolution of 2048 × 2048, the interpolation network runs for ~5 min on a machine with an AMD EPYC 7543P 32-Core Processor and two NVIDIA A100 80 GB GPU cards. In terms of memory limitations, we could not fit the full unbinned data into memory. Therefore, we ran all DL interpolation tests on data binned by a factor of 2, which corresponds to the aforementioned resolution.

### Statistics and reproducibility

Data were presented as boxplots showing the median, interquartile range (IQR), and whiskers extending to 1.5× IQR. Outliers and all individual data points are overlaid as dots. Statistical comparisons were performed using either the two-sided Wilcoxon signed-rank test or the paired samples *t*-test, as specified in each figure, together with the sample size. Statistical significance is indicated as follows: ***p < 0.001, **p < 0.01, *p < 0.05, and “ns” for not significant.

### Reporting summary

Further information on research design is available in the Nature Portfolio Reporting Summary linked to this article.

## Supplementary information

Supplementary information

Description of Additional Supplementary Files

Supplementary Data 1

Supplementary Data 2

Supplementary Data 3

Supplementary Data 4

Supplementary Data 5

Reporting Summary

Transparent Peer Review file

## Data Availability

The datasets used to train models are part of ongoing research and are therefore not publicly available. The primary data from the ribosome and NPC NR evaluation are publicly available on EMPIAR (EMPIAR-11899 and EMPIAR-12454, respectively). The tomograms used for DL-based segmentation are available at the CZI data portal—the dataset ID is CZCDP-10004, and tilt series 128_2, 129_2, 133, and 141_3 were used for evaluation. The primary data from the nucleosome study will be publicly available once the study is peer-reviewed and published. The data points underlying the graphs are provided as supplementary files: Supplementary Data 1 (Fig. 2), Supplementary Data 2 (Fig. 3), Supplementary Data 3 (Fig. 4), Supplementary Data 4 (Fig. 5), and Supplementary Data 5 (Figs. 6, 7).

## Code availability

The framework cryoTIGER is publicly available on GitHub https://github.com/turonova/cryoTIGER/ in the form of a Jupyter Notebook (under GPL-3.0 license). All trained models, as well as a minimal example on how to run the interpolation on tilt series, are provided there as well. The repository also includes *config.gin* file which includes all hyperparameters used in this study.
