# Supplementary material for: cryoTIGER: deep-learning based tilt interpolation generator for enhanced reconstruction in cryo electron tomography
Source: Commun Biol. 2025 Oct 9;8:1443. doi: 10.1038/s42003-025-08961-5 (PMC12511353; doi:10.1038/s42003-025-08961-5)
Supplement: Supplementary file 1 — Supplementary information [file 42003_2025_8961_MOESM1_ESM.pdf]

# Supplementary Information

## FFT artifacts during training of DL (Vimeo) model

When training DL cryo-ET model, we examined the Fourier transform (FFT) of the generated tilt images to visualize the presence of signals across different wavelengths. As a reference, we used a microscopic tilt image and its FFT (Supplementary Figure 4a), where no visible artifacts were observed. We then attempted to train the model using *D. discoideum* images with different data binning (Supplementary Figure 4b,c), a decreased learning rate (Supplementary Figure 4d), and transfer learning with weights from a DL (Vimeo) model (Supplementary Figure 4e). Additionally, we experimented with data from Human T cells (Supplementary Figure 4f) and extracted 256×256 patches from larger inputs (Supplementary Figure 4g,h). Each of these tests introduced specific artifacts in the frequency domain.

To better understand the causes of these distortions, we analyzed the original RGB Vimeo-90k dataset[1] (Supplementary Figure 5a) and applied a series of modifications to observe their effects in the frequency domain. Converting the RGB inputs to grayscale (Supplementary Figure 5b) and adding artificial CTF deformation (Supplementary Figure 5c) produced no visible differences. However, introducing Gaussian noise to the Vimeo-90k dataset (Supplementary Figure 5d) resulted in FFT artifacts similar to those observed in cryo-ET data. This led us to conclude that the primary factor causing artifacts in cryo-ET data is the presence of noise, likely in combination with other factors.

## Details on image comparison metrics

To assess the similarity between images, we used PSNR, RMSE, and SSIM metrics. The PSNR is a measure used to assess the quality of a reconstructed image compared to the original image. It represents the ratio between the maximum achievable power of a signal and the power of noise that disrupts its accuracy. Given that many signals span a broad dynamic range, PSNR is typically measured on a logarithmic scale in decibels (dB). PNSR is given by the formula:

$$\mathrm{PSNR} = 10 \cdot \log_{10}\left(\frac{\mathrm{MAX}^2}{\mathrm{MSE}}\right)$$

where MAX is the maximum possible pixel value of the image (e.g., 255 for 8-bit images) and MSE is the Mean Squared Error, calculated as:

$$\mathrm{MSE} = \frac{1}{m \cdot n}\sum_{i=1}^{m}\sum_{j=1}^{n}[I(i,j) - K(i,j)]^2$$

Here, $m$ and $n$ are the dimensions of the image, $I(i,j)$ is the pixel value of the original image at position $(i,j)$, and $K(i,j)$ is the pixel value of the reconstructed image at position $(i,j)$.

A higher PSNR value indicates better quality, with less distortion between the original and reconstructed images. PSNR is logarithmic, and a difference of 1 dB represents a multiplicative change in the MSE. Specifically, a difference of 1 dB indicates that the ratio of the peak signal power to the noise power has changed by approximately 26 %. This means the noise level in the image has either increased or decreased accordingly.

The RMSE is a metric used to quantify the difference between reconstructed values and actual values. It gives higher weight to larger errors, making it sensitive to outliers, and it is calculated as the square root of the average of the squared differences:

$$\mathrm{RMSE} = \sqrt{\mathrm{MSE}}$$

The RMSE is always non-negative, with lower values indicating smaller errors and better performance. RMSE directly measures the error, so a difference of 1 unit in RMSE means that, on average, the deviation between the processed and original image changes by 1 unit in the same scale as the data.

The SSIM is a metric used to assess the similarity between two images by focusing on structural information rather than pixel-wise differences. SSIM combines luminance, contrast, and structural comparisons into a single score ranging from $-1$ (completely dissimilar) to 1 (perfect similarity).

The formula for SSIM is:

$$\mathrm{SSIM}(I,K) = \frac{(2\mu_I\mu_K + C_1)(2\sigma_{IK} + C_2)}{(\mu_I^2 + \mu_K^2 + C_1)(\sigma_I^2 + \sigma_K^2 + C_2)}$$

where $\mu_I$ is the mean intensity of image $I$, $\mu_K$ is the mean intensity of image $K$, $\sigma_I^2$ is the variance of image $I$, $\sigma_K^2$ is the variance of image $K$, $\sigma_{IK}$ is the covariance of images $I$ and $K$, $C_1 = (K_1 \cdot L)^2$ is the stabilization constant for luminance, $C_2 = (K_2 \cdot L)^2$ is the stabilization constant for contrast, $L$ is the dynamic range of pixel values (e.g., 255 for 8-bit images), and $K_1$ and $K_2$ are small constants (default values: $K_1 = 0.01$, $K_2 = 0.03$).

SSIM considers the luminance (comparing the mean intensity values), contrast (comparing the variances of the images), and structure (comparing the covariance of the images). A higher SSIM value indicates greater similarity between the two images. A difference of 0.01 in SSIM (e.g., from 0.85 to 0.86) indicates a 1 % change in structural similarity and larger differences (e.g., from 0.70 to 0.80) reflect a substantial improvement in similarity.

## Details on metrics for evaluating peak selection

To assess the performance of particle selection, we computed the F1 score, precision, recall, and the PR-AUC between found positions and the GT lists.

To evaluate TM results with and without interpolation, we extracted peaks with different thresholds and compared them against baseline positions. When evaluating the match between a list of found peak positions and the baseline positions, we introduced a tolerance distance to account for imprecisions arising from particle list conversions. A peak was considered a match if it was found within this tolerance distance from the baseline position.

Precision measures the proportion of correctly predicted positive samples among all positive predictions. It is defined as:

$$\text{Precision} = \frac{\text{TP}}{\text{TP} + \text{FP}}$$

where TP are True Positives (correctly predicted positive samples) and FP are False Positives (incorrectly predicted positive samples).

Recall (also known as Sensitivity) measures the proportion of correctly predicted positive samples among all actual positive samples. It is defined as:

$$\text{Recall} = \frac{\text{TP}}{\text{TP} + \text{FN}}$$

where FN are False Negatives (actual positives incorrectly predicted as negative).

The F1 Score is the harmonic mean of precision and recall, providing a single measure that balances the two metrics. It is defined as:

$$\text{F1 Score} = 2 \cdot \frac{\text{Precision} \cdot \text{Recall}}{\text{Precision} + \text{Recall}}$$

The Precision-Recall AUC is the area under the precision-recall curve, which plots precision ($y$-axis) against recall ($x$-axis) for different thresholds. It is computed as:

$$\text{PR-AUC} = \int_0^1 \text{Precision}(\text{Recall})\, d(\text{Recall})$$

Here, the integral is calculated over the range of recall values from 0 to 1.

## Details on metrics for evaluating segmentation

To quantitatively assess the quality of image segmentation results, we used three widely used metrics: the Jaccard Index, the Dice Coefficient, and the Hausdorff Distance.

The Jaccard Index, also known as the Intersection over Union (IoU), measures the similarity between two sets: the predicted segmentation $A$ and the ground truth $B$. It is defined as the size of the intersection divided by the size of the union of the two sets:

$$J(A,B) = \frac{|A \cap B|}{|A \cup B|}$$

The value of $J(A,B)$ ranges from 0 (no overlap) to 1 (perfect overlap).

The Dice Coefficient, also known as the Sørensen–Dice index, is another similarity measure between two sets. It is defined as twice the size of the intersection divided by the sum of the sizes of the two sets:

$$D(A,B) = \frac{2|A \cap B|}{|A| + |B|}$$

Like the Jaccard Index, the Dice Coefficient ranges from 0 to 1, where 1 indicates perfect agreement. The Dice Coefficient is related to the Jaccard Index via the formula:

$$D(A,B) = \frac{2J(A,B)}{1 + J(A,B)}$$

The Hausdorff Distance measures the maximum distance of a point in one set to the nearest point in the other set. For two sets of points $A$ and $B$, the directed Hausdorff distance from $A$ to $B$ is defined as:

$$h(A,B) = \max_{a \in A} \min_{b \in B} \|a - b\|$$

This metric captures the worst-case mismatch between the boundaries of the two segmentations, making it sensitive to outliers. It is good for detecting boundary errors or outliers that the previous two metrics might miss.

| # of TS | Tilt increment | # of triplets | # of iterations | Organism (image proc. method) |
|---|---|---|---|---|
| **375** | **1+2+3** | **317,312** | **up to 3mil** | **All from Table 1** |
| 285 | 1+2+3 | 14,987 | up to 2mil | *D. discoideum* (bin4 data) |
| 285 | 1+2+3 | 239,791 | up to 2mil | *D. discoideum* (patches from bin4 data) |
| 306 | 3 | 166,175 | up to 5mil | *D. discoideum* (patches from bin4 data) |
| 52 | 1 | 5,318 | up to 5mil | *D. discoideum* (bin4 data) |
| 119 | 2 | 6,665 | up to 5mil | *D. discoideum* (bin4 data) |
| 159 | 3 | 5,678 | up to 5mil | *D. discoideum* (bin4 data) |
| 171 | 1+2 | 12,343 | up to 5mil | *D. discoideum* (bin4 data) |
| 32 | 2 | 28,976 | up to 1mil | Human T cells (bin4 data) |
| 32 | 2 | 66,996 | up to 1mil | Human T cells (patches from bin4 data) |
| 32 | 2 | 66,996 | up to 3mil | Human T cells (deconvolved) |
| 32 | 2 | 66,996 | up to 3mil | Human T cells (Topaz[2]) |

**Supplementary Table 1.** Summary of tested models and configurations. Models with fewer iterations than specified were also stored during the training. The training was done either directly on bin4 data or on 256×256 patches as described in the main text. The deconvolution for the Human T cells was performed using deconvolution function from the tiltstack module from cryoCAT.

| Analysis | Source | ID |
|---|---|---|
| Ribosomes | EMPIAR | EMPIAR-11899 |
| NPCs | EMPIAR | EMPIAR-12454 |
| Nucleosomes | Kreysing and Cruz-Leon et al.[3] | Not available |
| Membrane | CZI data portal | CZCDP-10004 (TS: 128_2, 129_2, 133, 141_3) |
| Microtubules | Kreysing and Cruz-Leon et al.[3] | Not available |

**Supplementary Table 2.** Summary of datasets used for presented analysis.

| Defocus diff | | | | | |
|---|---|---|---|---|---|
| Method | Count | Mean | Std | Vs Method | Paired t-test p-value |
| DL (Vimeo) | 111 | 0.3101 | 0.5123 | DL (cryo-ET) | 3.841011e-01 |
| DL (cryo-ET) | 111 | 0.2746 | 0.4127 | DL (Vimeo) | 3.841011e-01 |
| DL (Vimeo) | 111 | 0.3101 | 0.5123 | Linear | 7.270744e-05 |
| Linear | 111 | 0.1170 | 0.2603 | DL (Vimeo) | 7.270744e-05 |
| DL (cryo-ET) | 111 | 0.2746 | 0.4127 | Linear | 5.415679e-06 |
| Linear | 111 | 0.1170 | 0.2603 | DL (cryo-ET) | 5.415679e-06 |
| **PSNR** | | | | | |
| Method | Count | Mean | Std | Vs Method | Paired t-test p-value |
| DL (Vimeo) | 367 | 29.5833 | 4.1078 | DL (cryo-ET) | 1.971744e-35 |
| DL (cryo-ET) | 367 | 28.8185 | 4.2144 | DL (Vimeo) | 1.971744e-35 |
| DL (Vimeo) | 367 | 29.5833 | 4.1078 | Linear | 1.524221e-274 |
| Linear | 367 | 28.5520 | 4.2657 | DL (Vimeo) | 1.524221e-274 |
| DL (cryo-ET) | 367 | 28.8185 | 4.2144 | Linear | 8.621155e-07 |
| Linear | 367 | 28.5520 | 4.2657 | DL (cryo-ET) | 8.621155e-07 |
| **RMSE** | | | | | |
| Method | Count | Mean | Std | Vs Method | Paired t-test p-value |
| DL (Vimeo) | 367 | 115.4284 | 129.9039 | DL (cryo-ET) | 1.382685e-13 |
| DL (cryo-ET) | 367 | 154.6079 | 218.6361 | DL (Vimeo) | 1.382685e-13 |
| DL (Vimeo) | 367 | 115.4284 | 129.9039 | Linear | 3.643365e-33 |
| Linear | 367 | 153.0972 | 183.8088 | DL (Vimeo) | 3.643365e-33 |
| DL (cryo-ET) | 367 | 154.6079 | 218.6361 | Linear | 6.046145e-01 |
| Linear | 367 | 153.0972 | 183.8088 | DL (cryo-ET) | 6.046145e-01 |
| **SSIM** | | | | | |
| Method | Count | Mean | Std | Vs Method | Paired t-test p-value |
| DL (Vimeo) | 367 | 0.5404 | 0.2140 | DL (cryo-ET) | 6.708313e-26 |
| DL (cryo-ET) | 367 | 0.5508 | 0.2243 | DL (Vimeo) | 6.708313e-26 |
| DL (Vimeo) | 367 | 0.5404 | 0.2140 | Linear | 1.340066e-226 |
| Linear | 367 | 0.4880 | 0.2110 | DL (Vimeo) | 1.340066e-226 |
| DL (cryo-ET) | 367 | 0.5508 | 0.2243 | Linear | 1.896397e-141 |
| Linear | 367 | 0.4880 | 0.2110 | DL (cryo-ET) | 1.896397e-141 |

**Supplementary Table 3.** Extended information for boxplots in Figure 2.

**Ribosome (tilt restoration)**

| Method 1 | Method 2 | Statistic | p-value | Significance |
|---|---|---|---|---|
| Linear | Noint | 89.000 | 8.392e-02 | ns |
| Linear | DL (cryo-ET) | 0.000 | 1.192e-07 | *** |
| Linear | DL (Vimeo) | 1.000 | 2.384e-07 | *** |
| Noint | DL (cryo-ET) | 1.000 | 2.384e-07 | *** |
| Noint | DL (Vimeo) | 0.000 | 1.192e-07 | *** |
| DL (cryo-ET) | DL (Vimeo) | 142.000 | 8.334e-01 | ns |

**NPC NR (tilt restoration)**

| Method 1 | Method 2 | Statistic | p-value | Significance |
|---|---|---|---|---|
| Linear | Noint | 18.000 | 4.826e-04 | *** |
| Linear | DL (cryo-ET) | 16.000 | 3.223e-04 | *** |
| Linear | DL (Vimeo) | 55.000 | 6.372e-02 | ns |
| Noint | DL (cryo-ET) | 101.000 | 8.983e-01 | ns |
| Noint | DL (Vimeo) | 4.000 | 1.335e-05 | *** |
| DL (cryo-ET) | DL (Vimeo) | 19.000 | 5.856e-04 | *** |

**Ribosome (tilt addition)**

| Method 1 | Method 2 | Statistic | p-value | Significance |
|---|---|---|---|---|
| Linear | Noint | 10.000 | 5.126e-06 | *** |
| Linear | DL (cryo-ET) | 0.000 | 1.192e-07 | *** |
| Linear | DL (Vimeo) | 0.000 | 1.192e-07 | *** |
| Noint | DL (cryo-ET) | 34.000 | 4.299e-04 | *** |
| Noint | DL (Vimeo) | 75.000 | 3.148e-02 | * |
| DL (cryo-ET) | DL (Vimeo) | 25.000 | 1.076e-04 | *** |

**NPC NR (tilt addition)**

| Method 1 | Method 2 | Statistic | p-value | Significance |
|---|---|---|---|---|
| Linear | Noint | 50.000 | 3.999e-02 | * |
| Linear | DL (cryo-ET) | 38.000 | 1.069e-02 | * |
| Linear | DL (Vimeo) | 102.000 | 9.273e-01 | ns |
| Noint | DL (cryo-ET) | 82.000 | 4.091e-01 | ns |
| Noint | DL (Vimeo) | 32.000 | 4.860e-03 | ** |
| DL (cryo-ET) | DL (Vimeo) | 10.000 | 8.202e-05 | *** |

**Supplementary Table 4.** Wilcoxon signed-rank test results comparing interpolation methods for each dataset. Sample size n = 24 for ribosome data and n = 20 for NPC NR. Significance levels: *** for $p < 0.001$, ** for $p < 0.01$, * for $p < 0.05$, and "ns" for non-significant.

**DeePiCt Ribosome picking PR-AUC statistics**

| Method 1 | Method 2 | Statistic | p-value | Significance |
|---|---|---|---|---|
| Linear | Noint | 53.000 | 4.335e-03 | ** |
| Linear | DL (cryo-ET) | 145.000 | 8.996e-01 | ns |
| Linear | DL (Vimeo) | 58.000 | 7.189e-03 | ** |
| Noint | DL (cryo-ET) | 47.000 | 2.246e-03 | ** |
| Noint | DL (Vimeo) | 0.000 | 1.192e-07 | *** |
| DL (cryo-ET) | DL (Vimeo) | 48.000 | 2.516e-03 | ** |

**DeePiCt Ribosome picking Max F1 score statistics**

| Method 1 | Method 2 | Statistic | p-value | Significance |
|---|---|---|---|---|
| Linear | Noint | 95.000 | 1.208e-01 | ns |
| Linear | DL (cryo-ET) | 129.000 | 5.646e-01 | ns |
| Linear | DL (Vimeo) | 25.000 | 1.076e-04 | *** |
| Noint | DL (cryo-ET) | 52.000 | 3.901e-03 | ** |
| Noint | DL (Vimeo) | 1.000 | 2.384e-07 | *** |
| DL (cryo-ET) | DL (Vimeo) | 10.000 | 5.126e-06 | *** |

**Supplementary Table 5.** Wilcoxon signed-rank test results comparing interpolation methods for DeePiCt Ribosome picking PR-AUC and Max F1 score. Sample size n = 24. Significance levels: *** for $p < 0.001$, ** for $p < 0.01$, * for $p < 0.05$, and "ns" for non-significant.

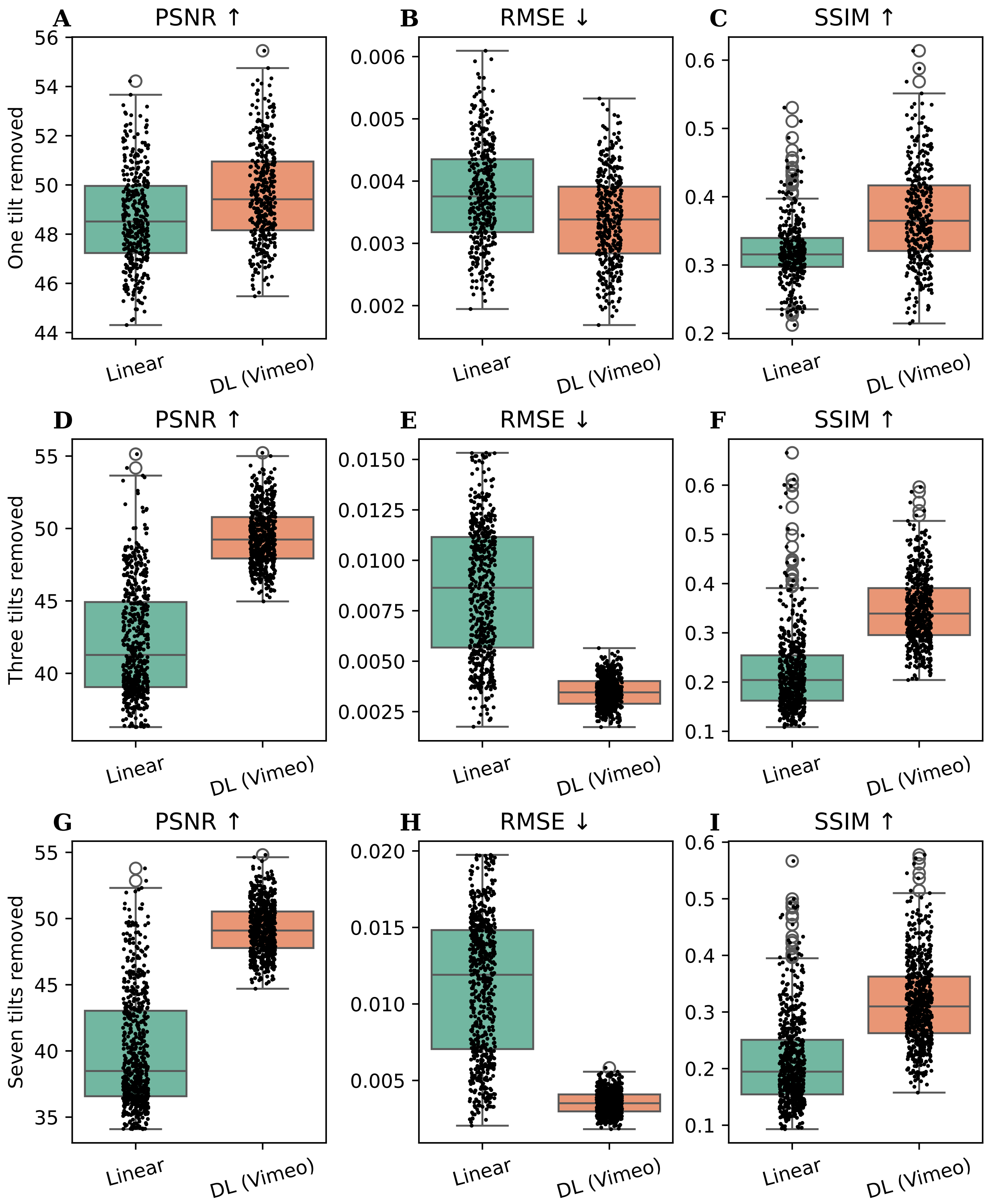


**Supplementary Figure 1.** Comparison of different interpolation methods when real tilts are removed. The test was done on the same data as Figure 2. Each boxplot contains data from seven tilt series binned by a factor of 8. Sample size n = 364 for A, B, C; n = 546 for D, E, F; n = 637 for G, H, I. Boxplots show medians, interquartile ranges (IQR), and whiskers up to 1.5× IQR; outliers and all individual values are overlaid as points.

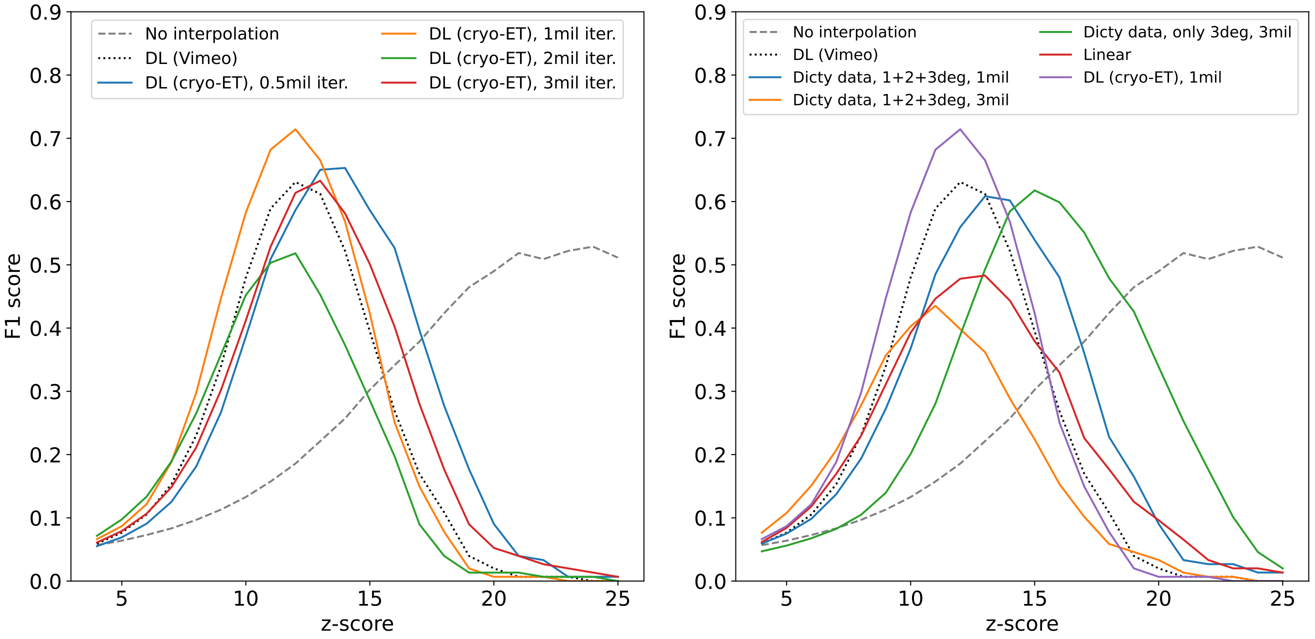


**(a)** The model training with a different number of iterations.

**(b)** Comparison of *DL (cryo-ET) model* with other selected trained models.

**Supplementary Figure 2.** Particle picking results for 80S ribosome on different trained models.

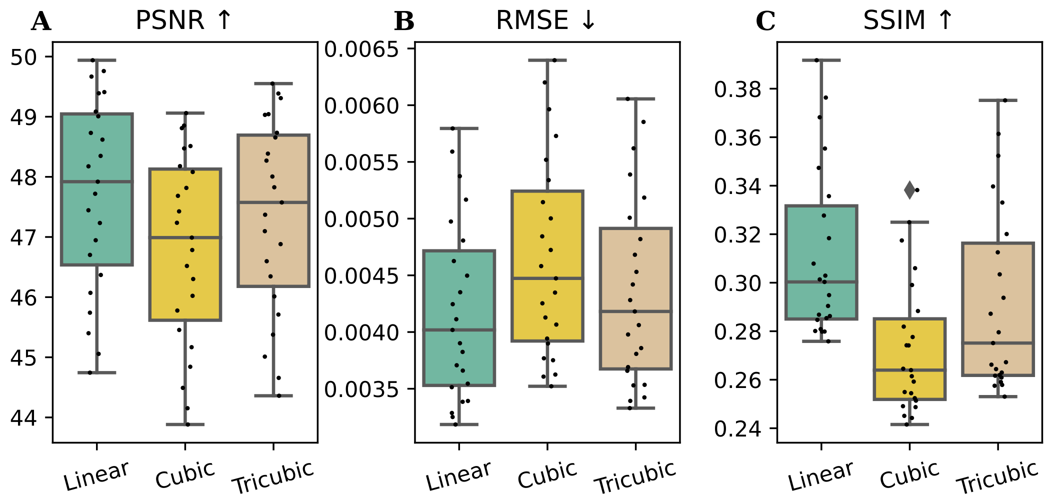


**Supplementary Figure 3.** Comparison between linear, cubic, and tricubic interpolation showing the best results for linear interpolation in all three metrics. Sample size n = 23. Boxplots show medians, interquartile ranges (IQR), and whiskers up to 1.5× IQR; outliers and all individual values are overlaid as points.

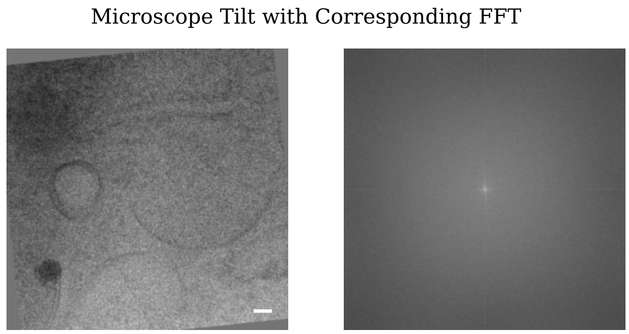


**(a)** Reference microscopic tilt image.

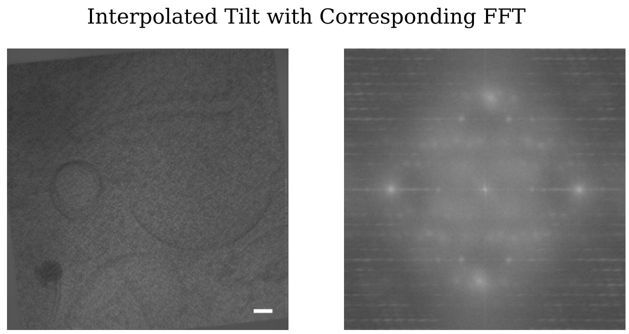


**(b)** Training on *D. discoideum*, bin4 input.

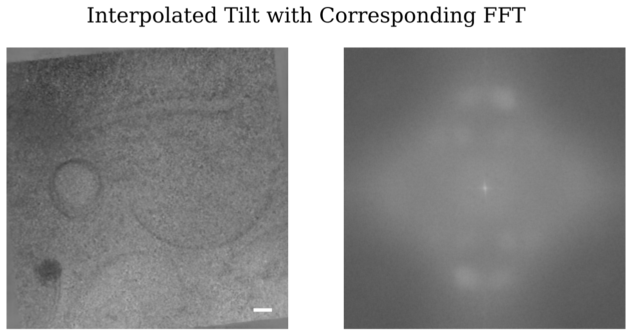


**(c)** Training on *D. discoideum*, bin16 input.

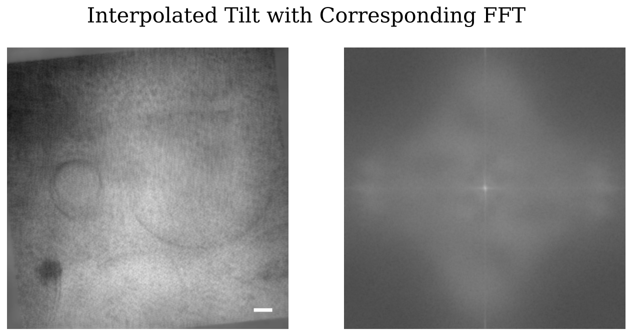


**(d)** Training on *D. discoideum*, decreased learning rate.

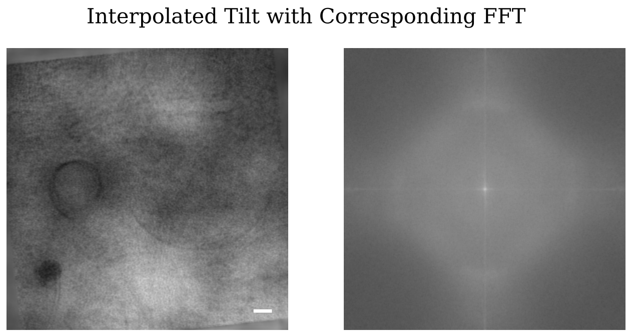


**(e)** Training on *D. discoideum*, transfer learning.

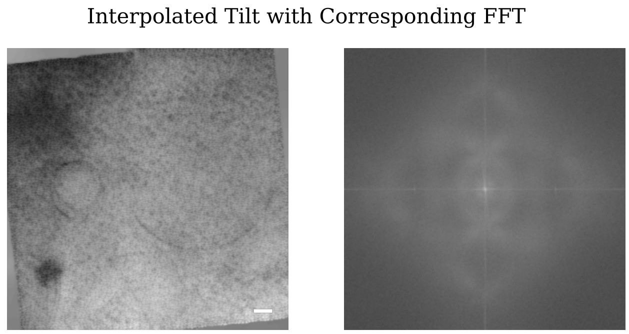


**(f)** Training on Human T cells, bin4 input.

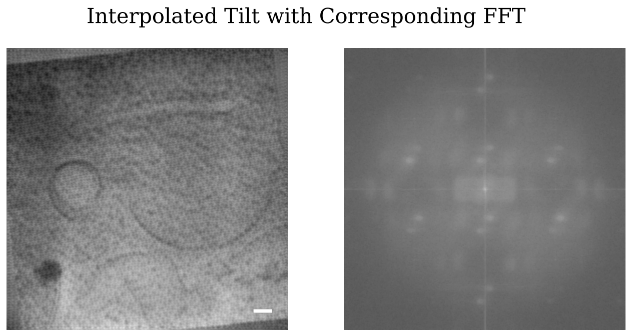


**(g)** Training on Human T cells, 256×256 patches.

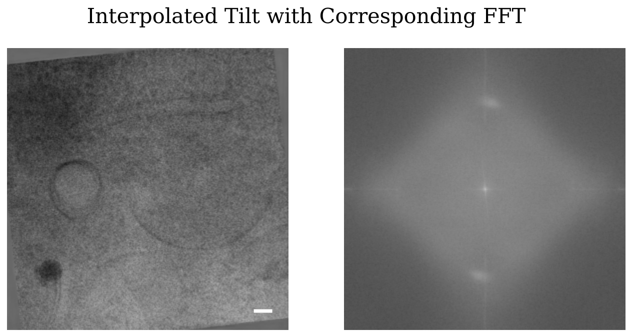


**(h)** Training on *D. discoideum*, 256×256 patches.

**Supplementary Figure 4.** The collection of FFT artifacts, when trained on cryo-ET data. Scale bar is 50 nm.

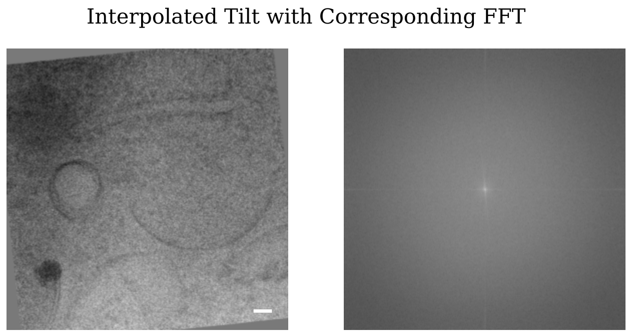


**(a)** Original RGB input in training.

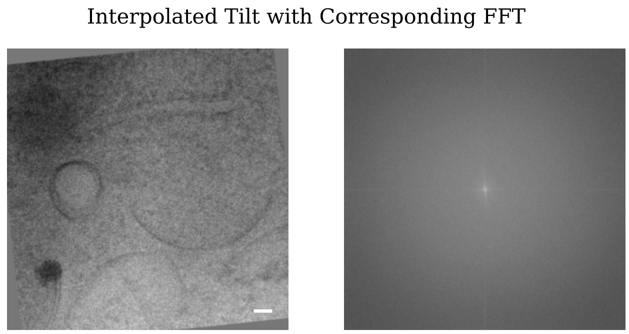


**(b)** Grayscale input in training.

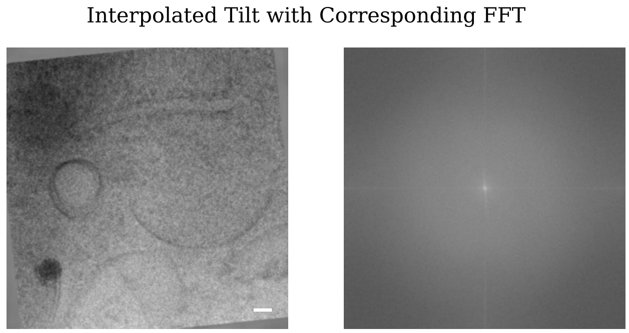


**(c)** Original RGB input plus CTF in training.

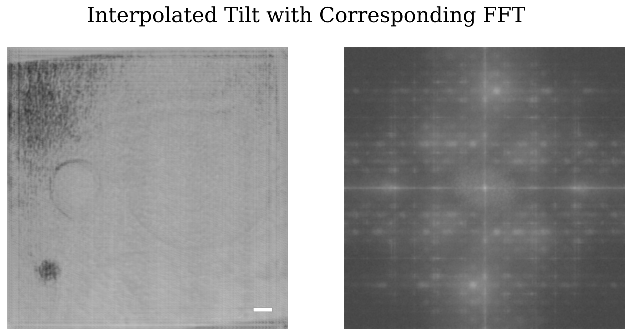


**(d)** Grayscale input plus Gaussian noise in training.

**Supplementary Figure 5.** The collection of FFT artifacts, when trained Vimeo-90k[1] dataset. Artifacts in training are the result of multiple factors, including noise. Scale bar is 50 nm.

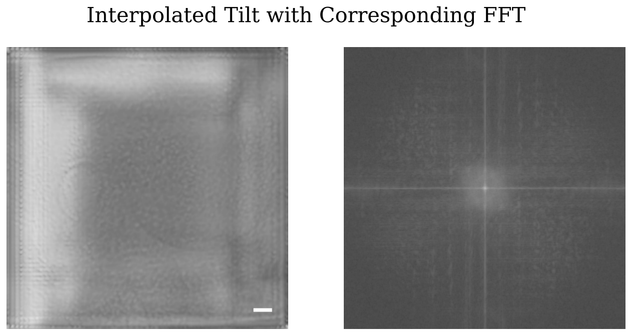


**(a)** Deconvolved input images.

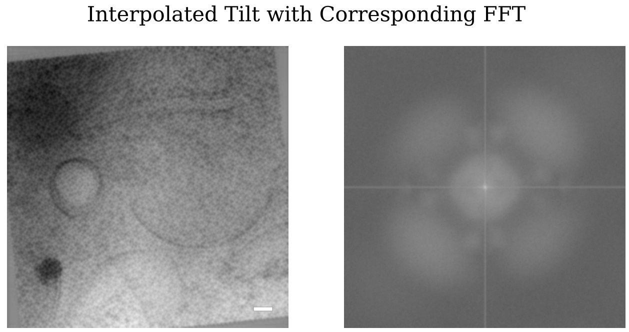


**(b)** Denoised input images using Topaz-Denoise[2].

**Supplementary Figure 6.** The collection of FFT artifacts, when trained on denoised images. Scale bar is 50 nm.

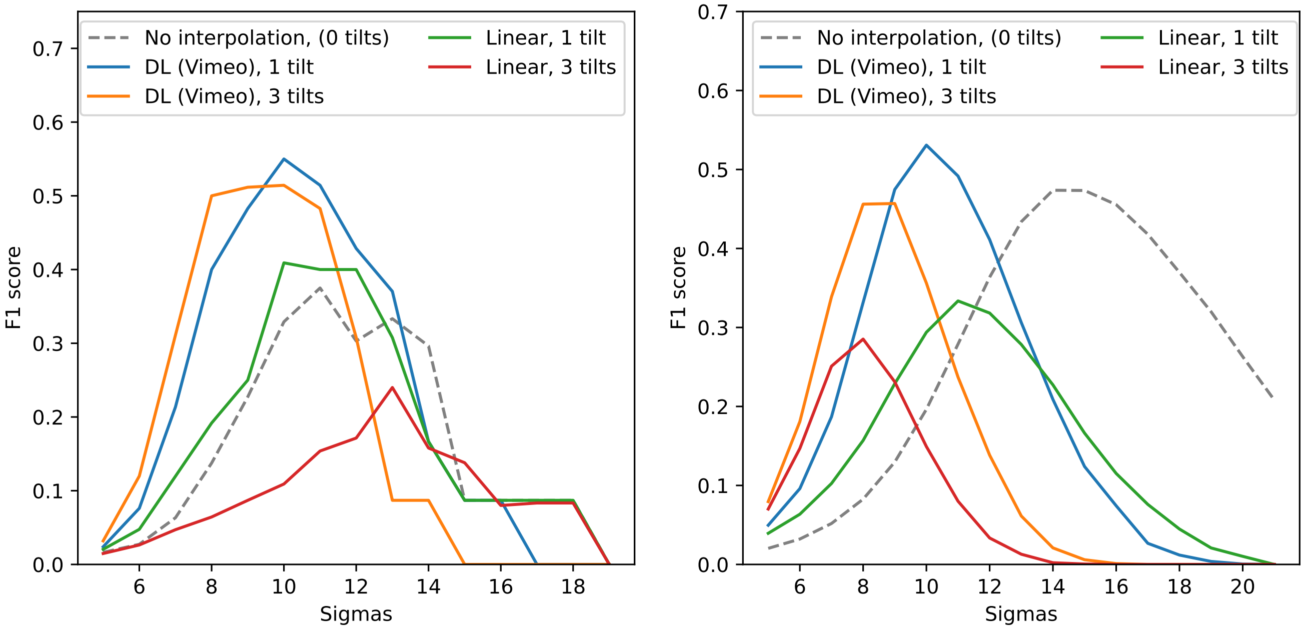


**(a)** NPC NR particle picking.

**(b)** Nucleosome particle picking.

**Supplementary Figure 7.** Comparison of adding one versus three interpolated tilts for different targets. For the nucleosome dataset, we compared the extracted list with the baseline, the formation of which is described in the main text. In both cases, using more interpolated tilts resulted in worse particle picking performance.

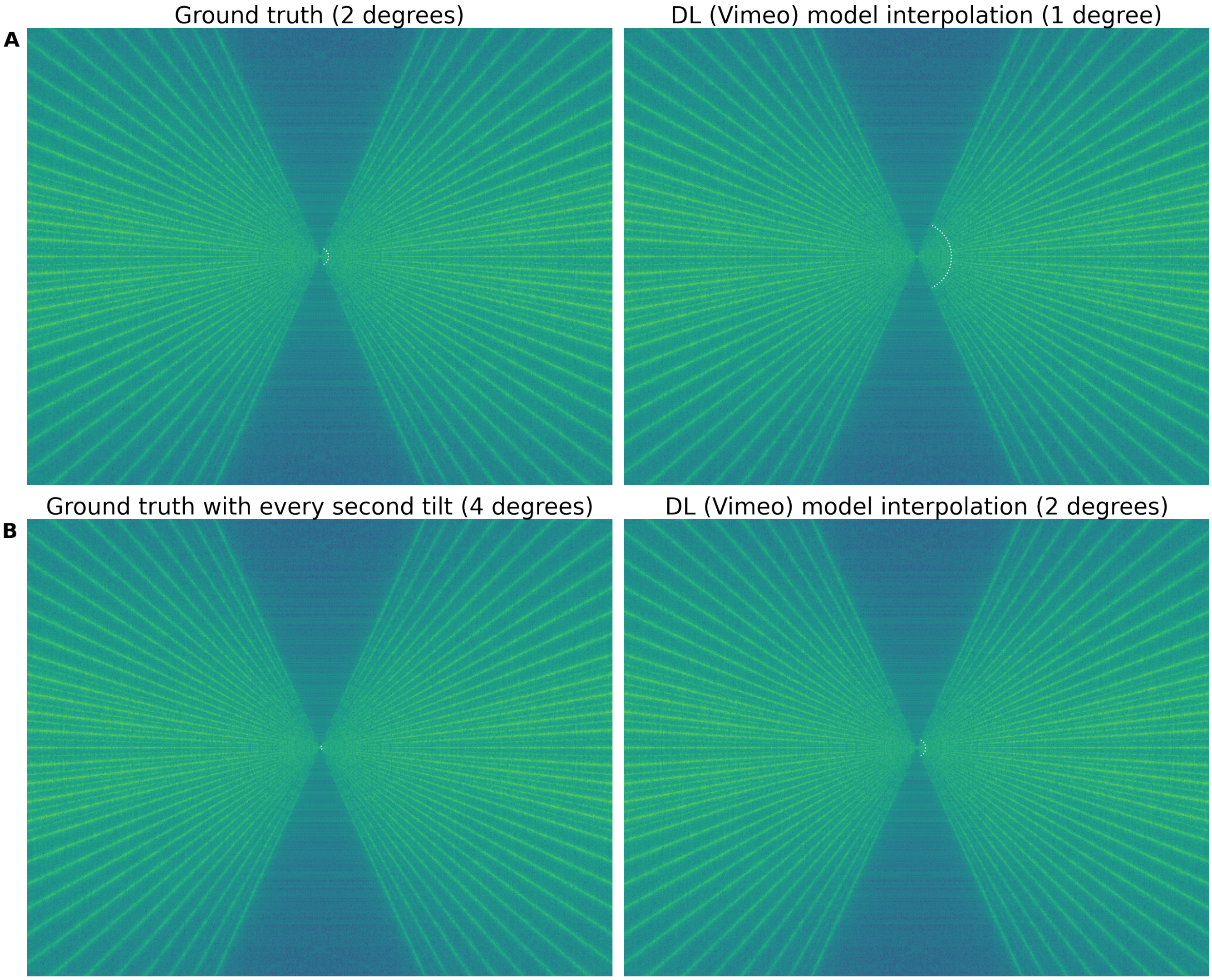


**Supplementary Figure 8.** Effects of the interpolation on angular sampling in the reconstructed tomogram. The white dotted sections mark the low-resolution area where the angular sampling is still complete. The radii in Fourier pixels were computed using the Crowther criterion formula. (A, left): Power spectrum of XZ tomogram slice of a reconstructed tomogram using the experimental tilt series with 61 images and 2 degrees tilt step. The complete angular sampling ends at a radius of 15.25 (in Fourier pixels). (A, right): Power spectrum of the same tomogram slice but with additional tilts created by DL-based interpolation using the DL (Vimeo) model. The tilt series contains 121 images, the tilt step is 1 degree, and the complete angular sampling ends at 60.5 Fourier pixels. (B) Power spectrum of the same tomogram slice as in (A, left) with every second tilt removed. The tilt series contains 31 images, the tilting step is 4 degrees, and the complete angular sampling ends at 3.875 Fourier pixels. (B, right) Power spectrum of the same tomogram slice as in (A, left) with every second tilt removed and then restored using DL-based interpolation with the DL (Vimeo) model. The tilt series contains 61 images, the tilt step is 2 degrees, and the complete angular sampling ends at 15.25 Fourier pixels.

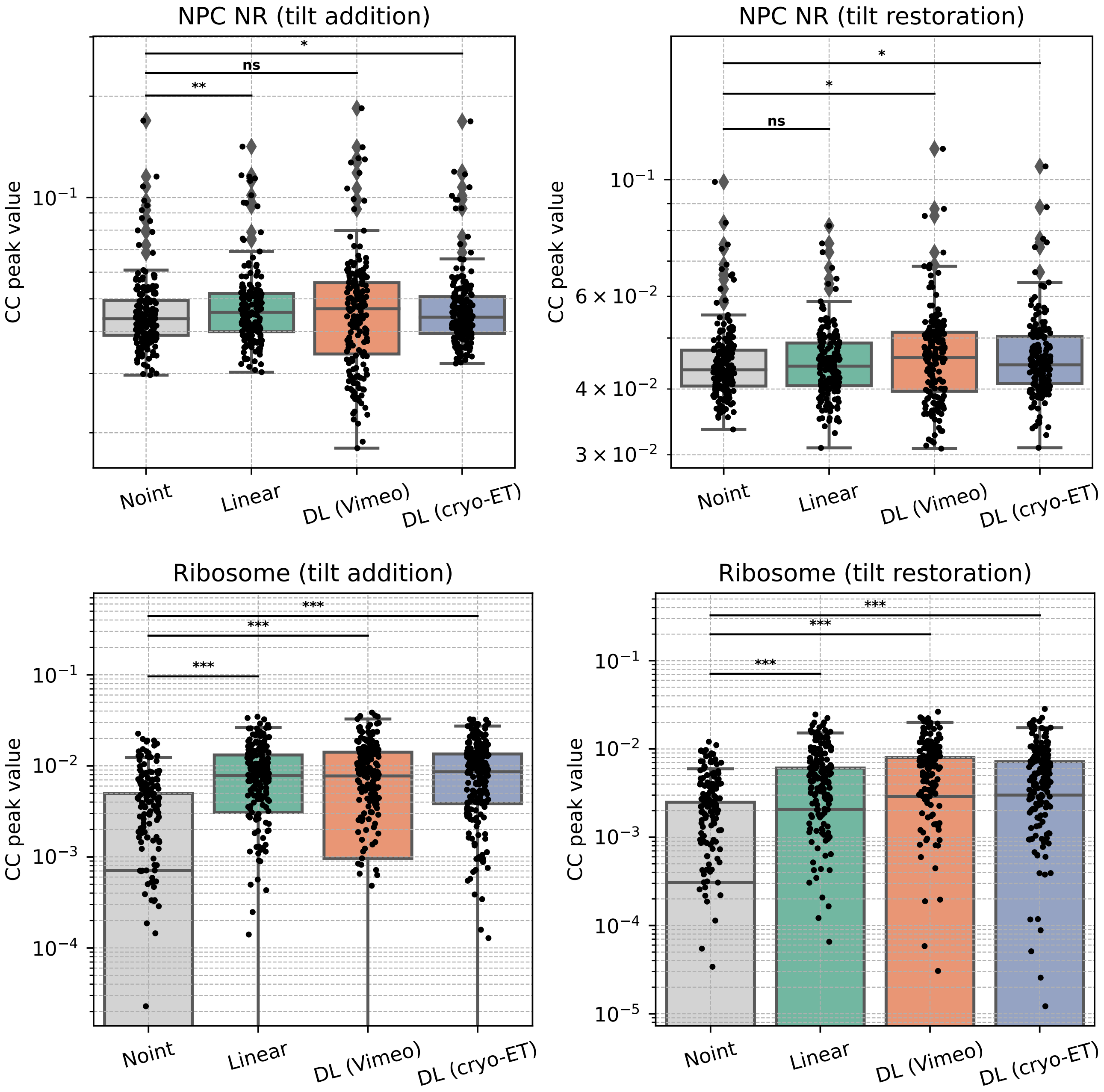


**Supplementary Figure 9.** NPC NR (first row) and 80S ribosome (second row) cross-correlation peak values. Pairwise group comparisons were assessed using the Wilcoxon signed-rank test (two-sided). Sample size n = 179 for NPC NR, n = 241 for ribosome data. Significance is indicated as *** for $p < 0.001$, ** for $p < 0.01$, * for $p < 0.05$, and "ns" for non-significant. Boxplots show medians, interquartile ranges (IQR), and whiskers up to 1.5× IQR; outliers and all individual values are overlaid as points.

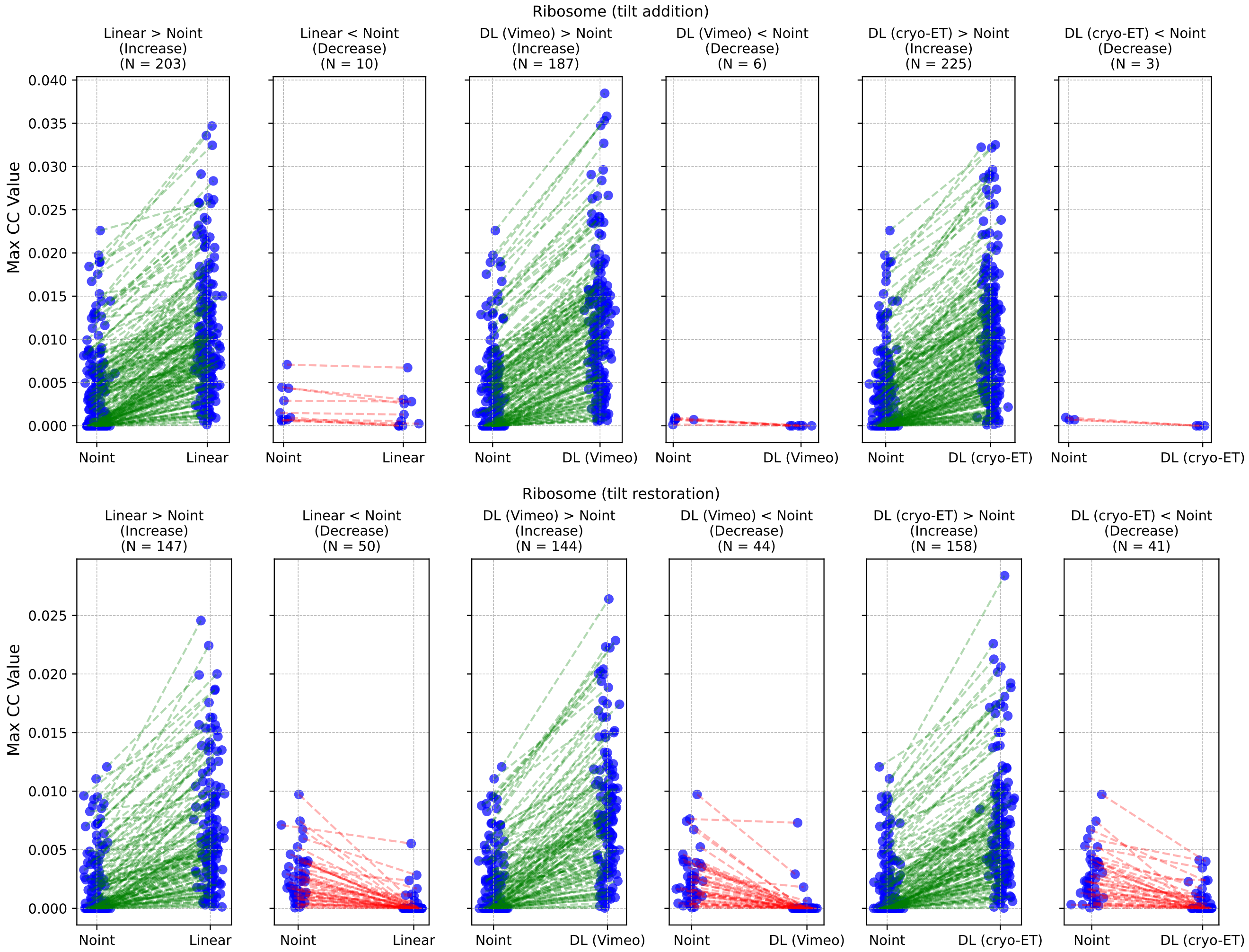


**Supplementary Figure 10.** Additional information to Supplementary Figure 9 connecting corresponding points and highlighting individual differences between maximum cross-correlation values for 80S ribosomes.

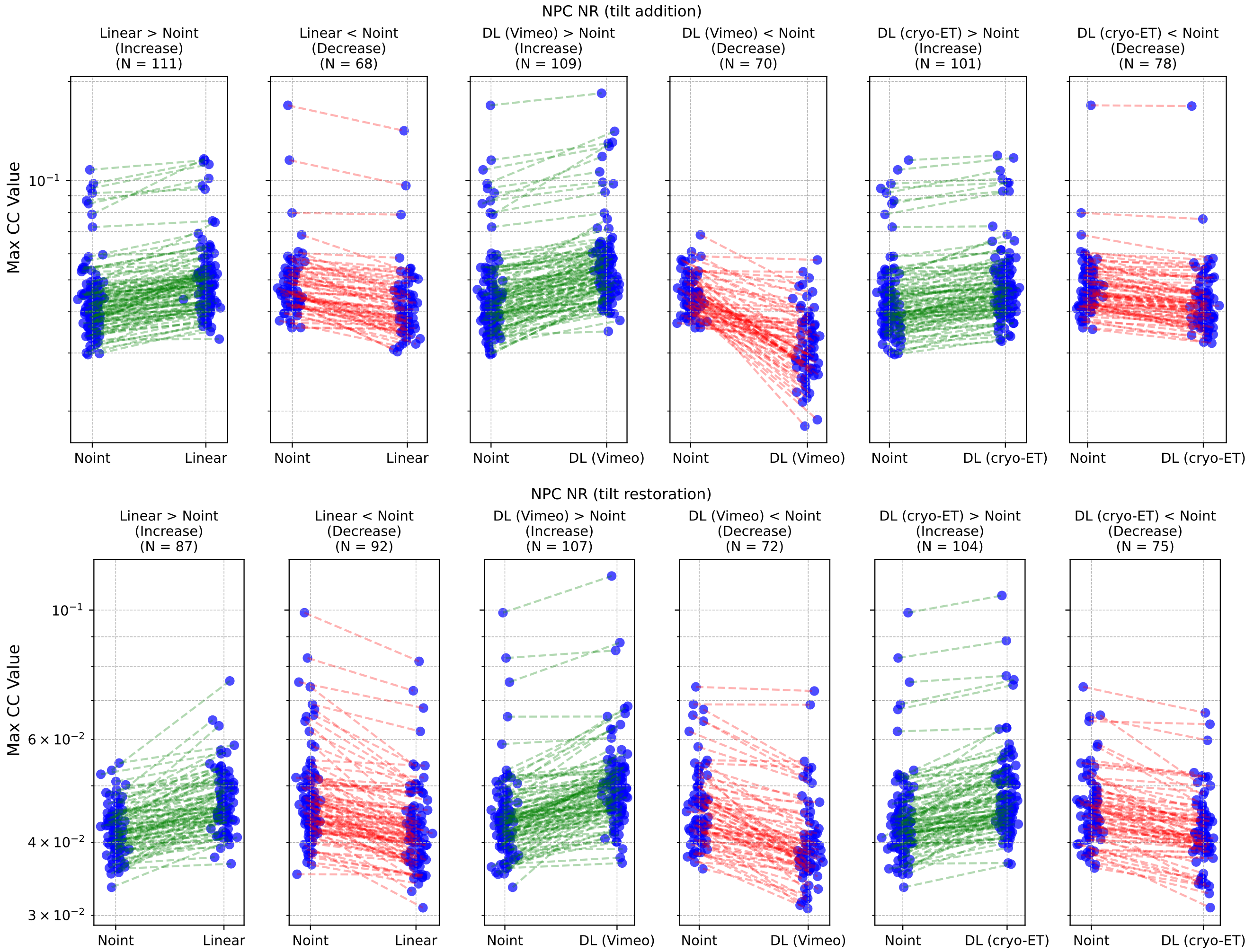


**Supplementary Figure 11.** Additional information to Supplementary Figure 9 connecting corresponding points and highlighting individual differences between maximum cross-correlation values for NPC NR.

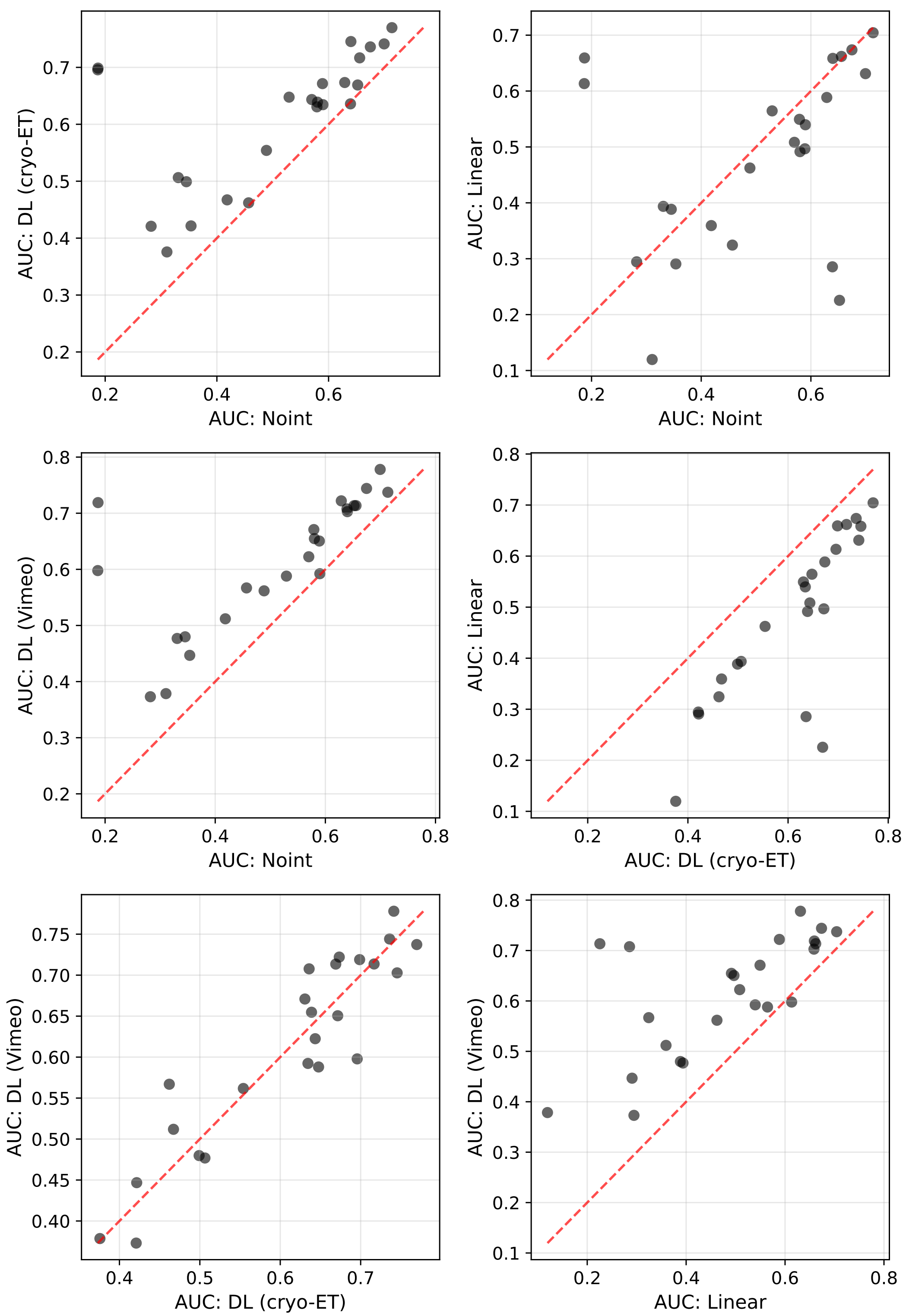


**Supplementary Figure 12.** All pairwise AUC comparisons for particle picking of ribosomes in tilt restoration condition using template matching. Supplement to Figure 3A. Sample size n = 24.

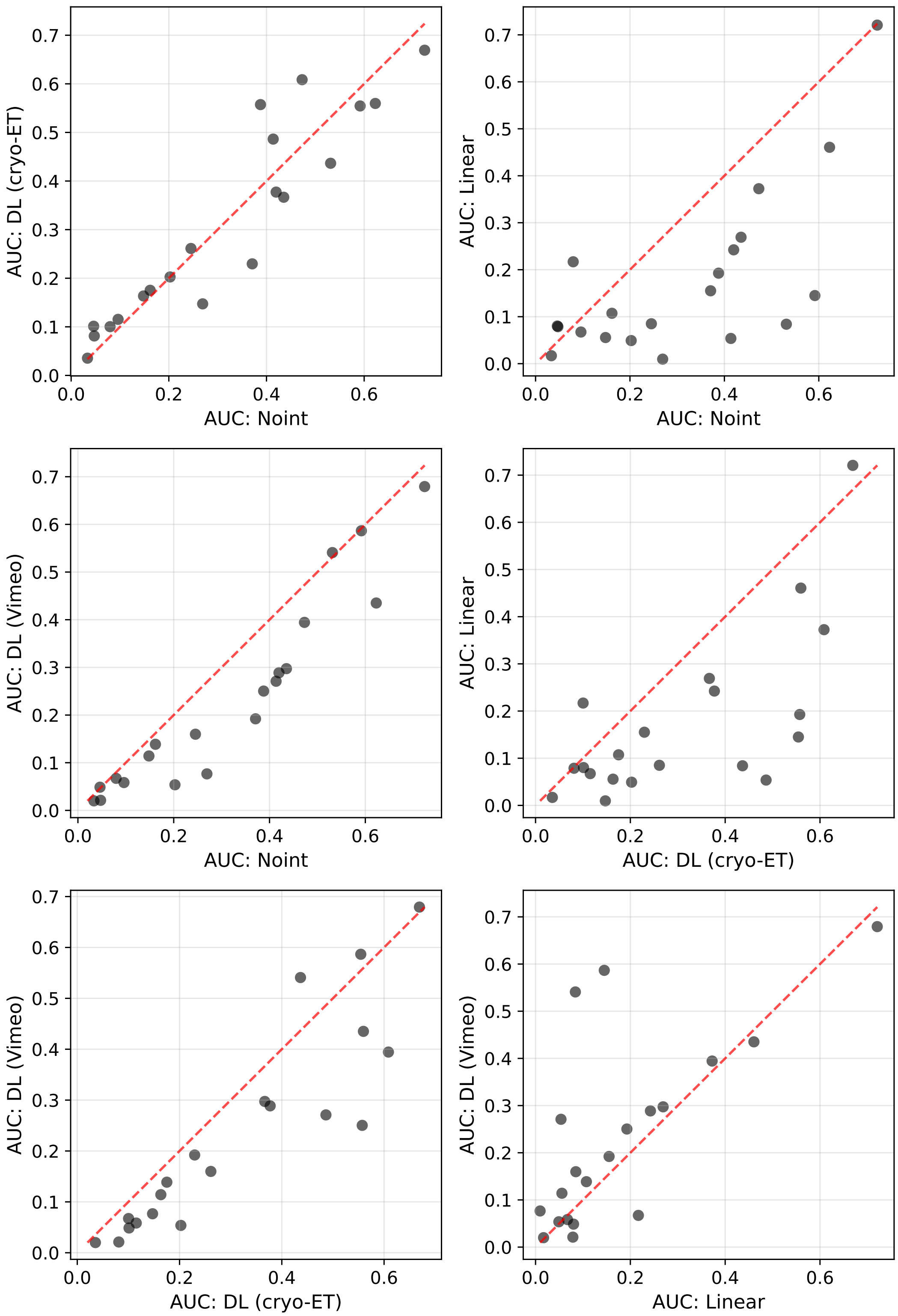


**Supplementary Figure 13.** All pairwise AUC comparisons for particle picking of NPC NR in tilt restoration condition using template matching. Supplement to Figure 3B. Sample size n = 20.

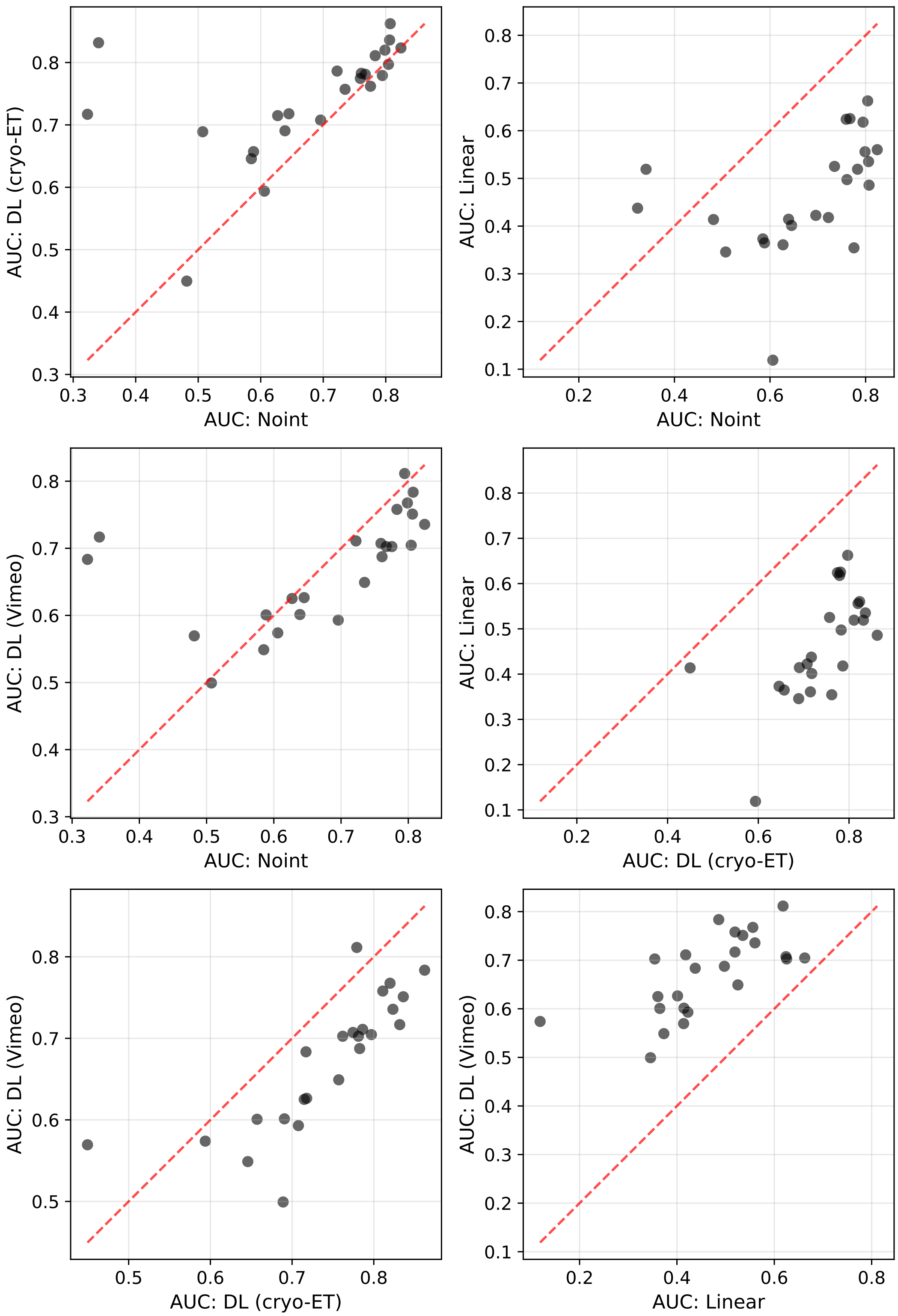


**Supplementary Figure 14.** All pairwise AUC comparisons for particle picking of ribosomes in tilt addition condition using template matching. Supplement to Figure 3C. Sample size n = 24.

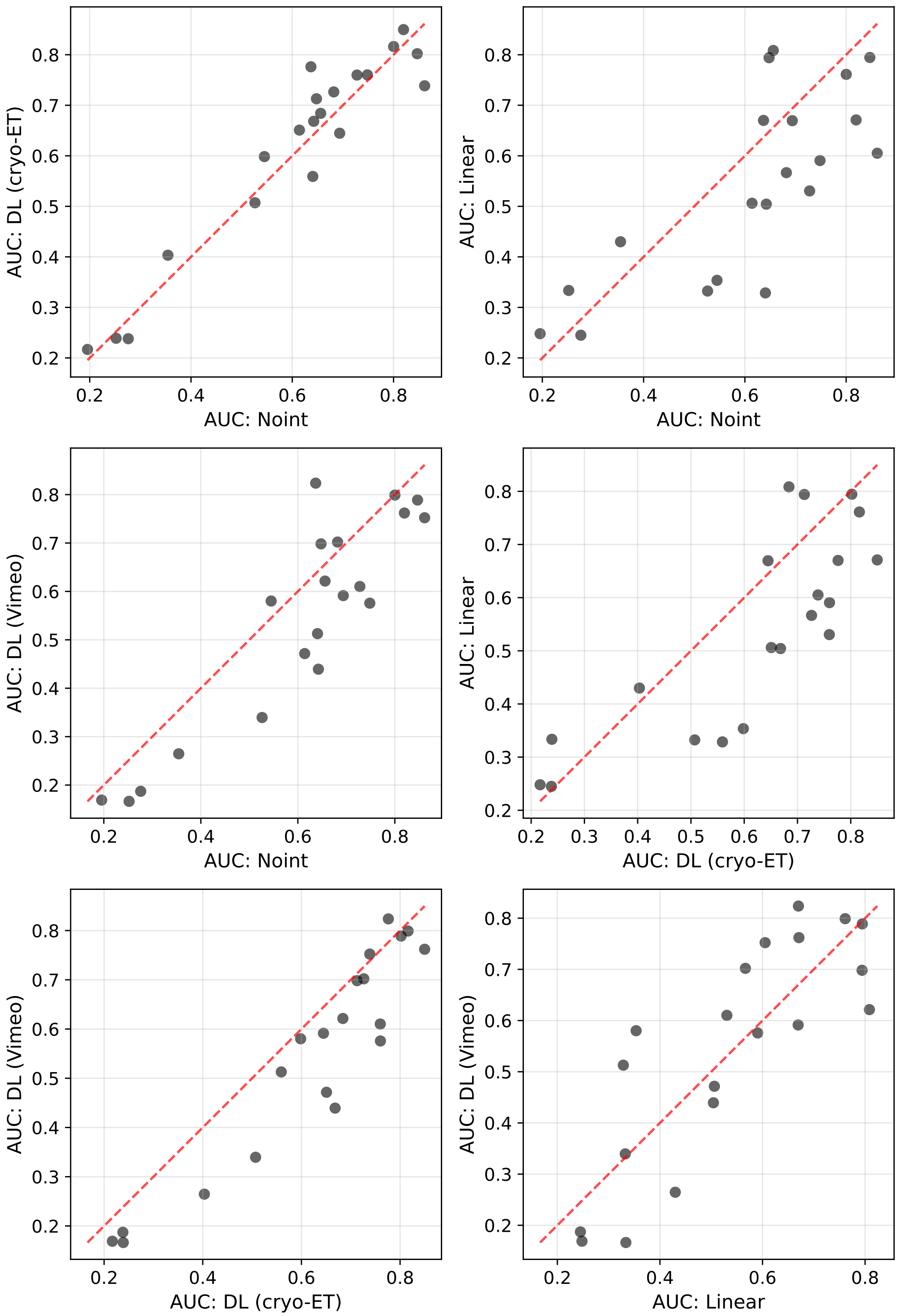


**Supplementary Figure 15.** All pairwise AUC comparisons for particle picking of NPC NR in tilt addition condition using template matching. Supplement to Figure 3D. Sample size n = 20.

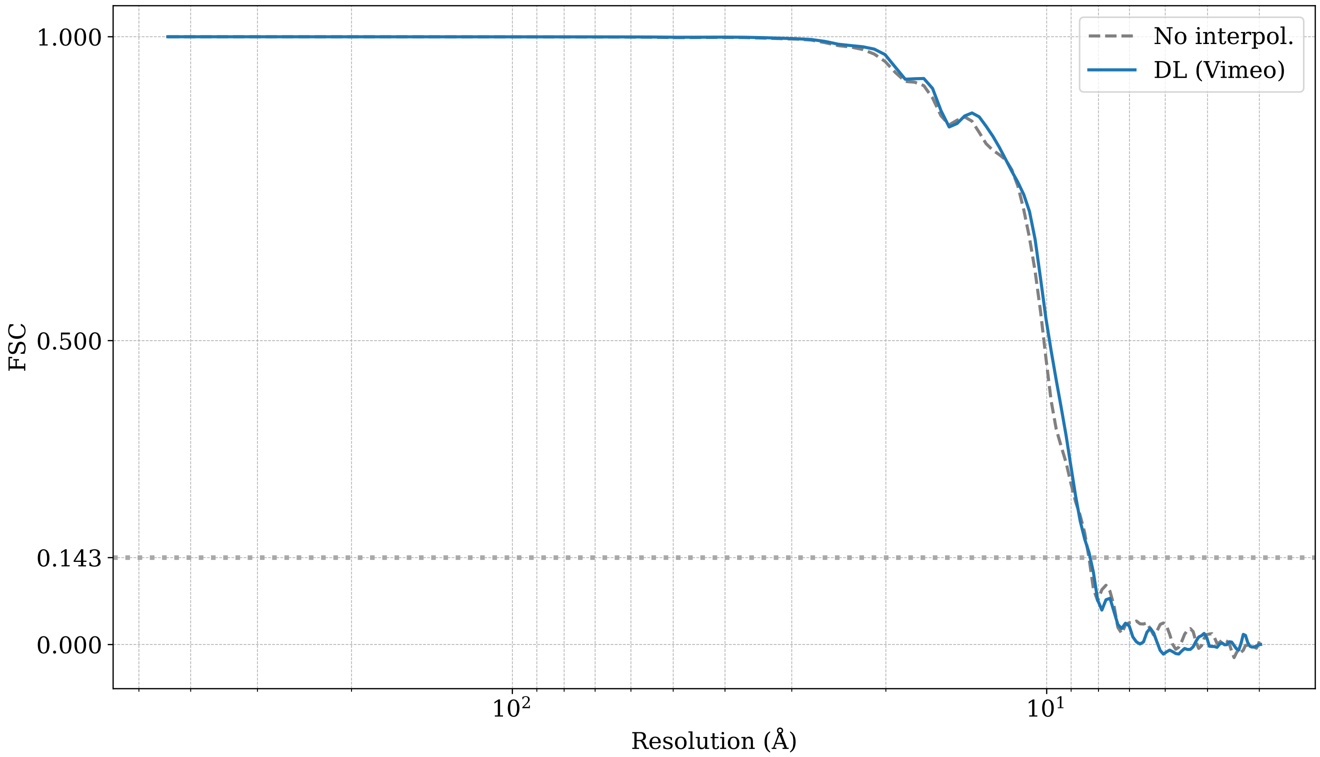


**Supplementary Figure 16.** Comparison of Fourier shell correlation (FSC) curves for a nucleosome structure determined using positions obtained from interpolated and non-interpolated data.

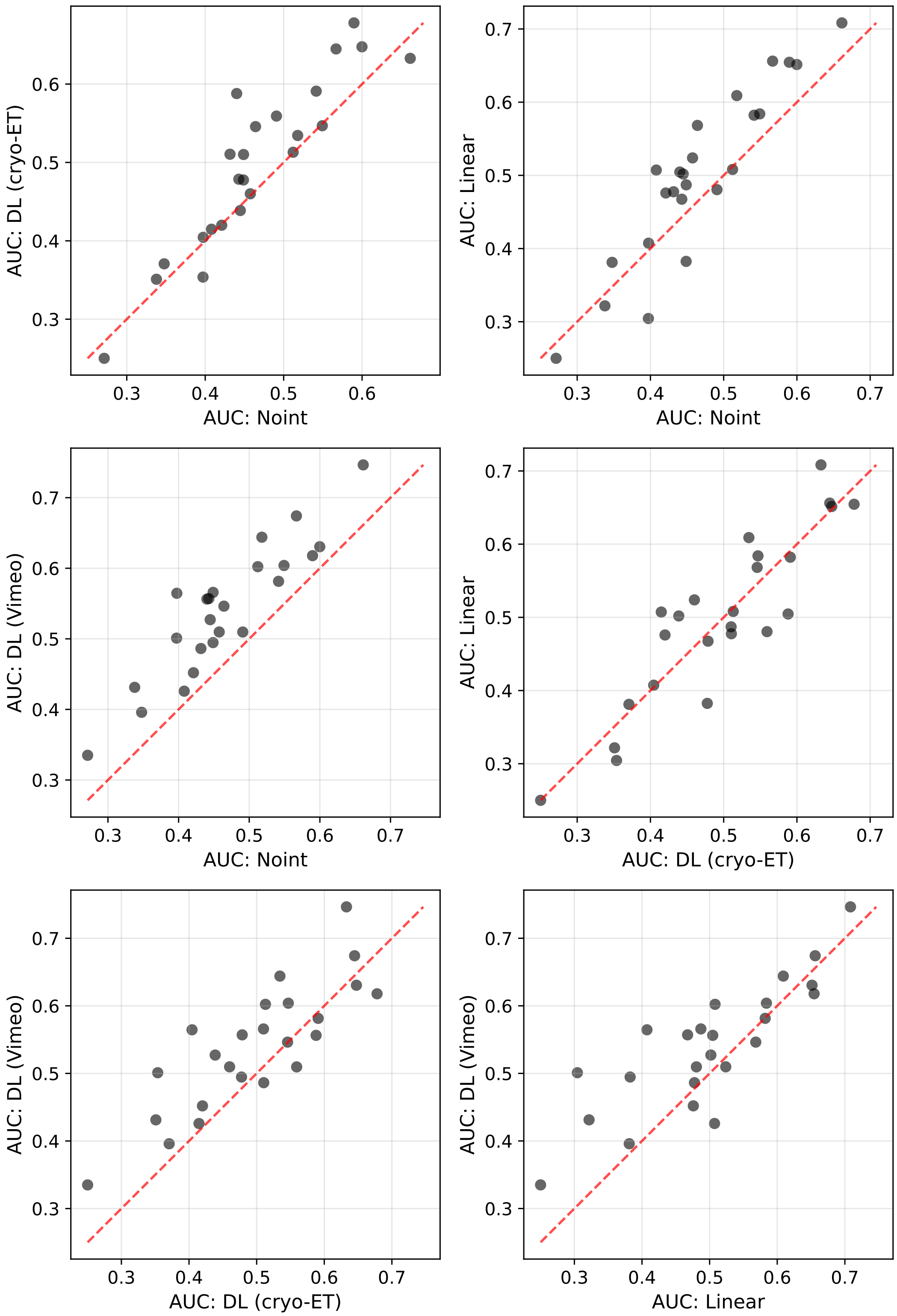


**Supplementary Figure 17.** All pairwise AUC comparisons for particle picking of ribosomes using DeePiCt. Supplement to Figure 5F. Sample size n = 24.

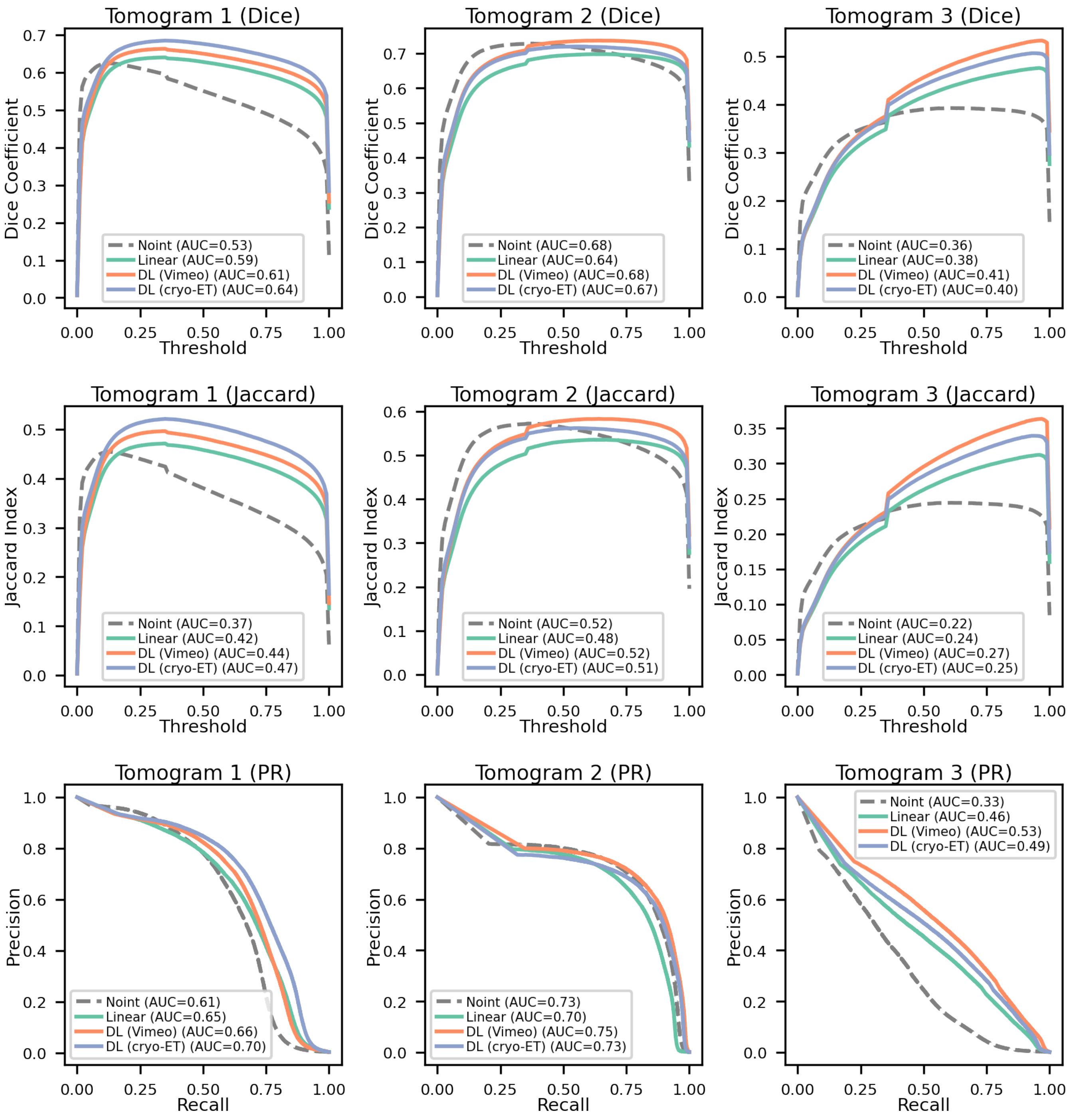


**Supplementary Figure 18.** Dice Coefficient and Jaccard Index curves across thresholds, and Precision-Recall (PR) curves, for microtubule segmentation using DeePiCt. Areas under each curve (AUC) are reported for quantitative comparison of methods. Supplement information to Figure 7.

## References


**1.** Xue, T., Chen, B., Wu, J., Wei, D. & Freeman, W. T. Video enhancement with task-oriented flow. *Int. J. Comput. Vis.* **127**, 1106–1125 (2019).

**2.** Bepler, T., Kelley, K., Noble, A. J. & Berger, B. Topaz-Denoise: general deep denoising models for cryoEM and cryoET. *Nat. Commun.* **11**, 5208 (2020).

**3.** Kreysing, J. P. *et al.* Molecular architecture of heterochromatin at the nuclear periphery of primary human cells. *bioRxiv* 2025–04 (2025).
