# Supplementary material for: cryoTIGER: deep-learning based tilt interpolation generator for enhanced reconstruction in cryo electron tomography
Source: Commun Biol. 2025 Oct 9;8:1443. doi: 10.1038/s42003-025-08961-5 (PMC12511353; doi:10.1038/s42003-025-08961-5)
Supplement: Supplementary file 2 — Description of Additional Supplementary Files [file 42003_2025_8961_MOESM2_ESM.docx]

**Description of Additional Supplementary Files**

**File name:** Supplementary Data 1

**Description:** Source file for graphs in figure 2.

**File name:** Supplementary Data 2

**Description:** Source file for graphs in figure 3.

**File name:** Supplementary Data 3

**Description:** Source file for graphs in figure 4.

**File name:** Supplementary Data 4

**Description:** Source file for graphs in figure 5.

**File name:** Supplementary Data 5

**Description:** Source file for graphs in figure 6 and 7.
